# A Study of Skeletal Stem Cell Dynamics and Its Potential Applications in the Design of a Titanium Implant for Senile Osteoporosis

**DOI:** 10.1002/advs.202506982

**Published:** 2025-06-19

**Authors:** Wuzhe Fan, Tao Zheng, Mingsong Mao, Pengfei Gao, Yulu Yang, Rong Wang, Yao Yang, Yangpeng Zuo, Tiantian Yuan, Ruqing Bai, Weihu Yang, Xingchen Yan, Kaiyong Cai

**Affiliations:** ^1^ Key Laboratory of Biorheological Science and Technology Ministry of Education College of Bioengineering Chongqing University Chongqing 400044 China; ^2^ School of Basic Medical Sciences Anhui Medical University Hefei 230032 China; ^3^ State Key Laboratory of Mechanical Transmission for Advanced Equipment Chongqing University Chongqing 400044 China; ^4^ Institute of New Materials Guangdong Academy of Sciences Guangdong‐Hong Kong Joint Laboratory of Modern Surface Engineering Technology Guangdong Provincial Key Laboratory of Modern Surface Engineering Technology Guangzhou Guangdong 510651 China

**Keywords:** anti‐aging, senile osteoporosis, single‐cell transcriptome sequencing, skeletal stem cells, Ti implant

## Abstract

Traditional biomaterial design often prioritizes empirical knowledge over disease mechanisms and pathological dynamics, resulting in imprecise solutions in complex clinical conditions. Age‐related osteoporosis (A‐OP) is a disease associated with aging, characterized by a dysfunctional pathological microenvironment that hinders the osseointegration of conventional titanium implants. To develop a targeted titanium implant for A‐OP, rat single‐cell transcriptomics is integrated with human serum‐derived transcriptome data to investigate dynamic changes in skeletal stem cells (SSCs) during aging, which guided the implant design. These findings reveal that hematopoietic stem cells (HSCs) and mesenchymal stem cells (MSCs) within SSCs interact via a feedback loop: HSCs undergo premature senescence, leading to depletion of HSCs and secondary senescence of MSCs. Senescent MSCs exhibit adipogenic bias, perpetuating the pathological cycle of A‐OP. Using core genes identified in the transcriptome analyses, resveratrol is selected and utilized it and a GelMA‐chitosan hydrogel to decorate titanium implants for localized delivery. In the A‐OP microenvironment, the hydrogel enables sustained responsive release of resveratrol, which reverses MSC senescence and redirects differentiation from adipogenic to osteogenic lineages, thereby breaking the pathological cycle. This multi‐omics‐driven implant design enhances precision and offers a novel methodology for biomaterial development.

## Graphical abstract

Senescent mesenchymal stromal cells (MSCs) drive age‐related osteoporosis (A‐OP) via adipogenic bias. Resveratrol, selected from transcriptome‐identified core genes, is delivered locally using GelMA‐chitosan hydrogel‐functionalised titanium implants. In A‐OP microenvironments, the hydrogel enables the sustained release of resveratrol, which reverses MSC senescence and redirects differentiation from the adipogenic to the osteogenic lineage. This breaks the pathological cycle of A‐OP.

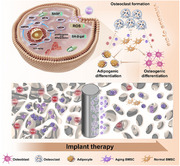

## 1 Introduction

Senile osteoporosis, a prevalent systemic metabolic bone disease affecting the elderly, is characterized by progressive bone loss and microstructural deterioration.^[^ ^1^ ^]^ Unlike other forms of osteoporosis (e.g., postmenopausal), its pathological microenvironment is more complex due to age‐related skeletal changes. These include DNA damage, epigenetic alterations, mitochondrial dysfunction, cellular senescence, stem cell depletion, and disrupted cellular communication. The aging skeleton undergoes a dynamic, irreversible process marked by progressive and multi‐step degeneration.^[^ ^2^ ^]^ Understanding these mechanisms could inform novel therapeutic strategies for senile osteoporosis and guide the development of specific bone repair materials.

Fractures and femoral head necrosis are common occurrences in patients with senile osteoporosis, and in some cases, a permanent internal fixation system or joint prostheses may be required. Titanium and its alloys are widely used as bone and dental implants due to their exceptional mechanical properties and biocompatibility.^[^ ^3^ ^]^ Recent research has revealed that titanium implants have a wider range of functions than previously thought.^[^ ^3^, ^4^ ^]^ However, the pathological microenvironment in senile osteoporosis differs from that in typical osteoporosis cases. Consequently, the osseointegration efficacy of Ti implants may be compromised if aging‐related factors are not considered during the design process.

The skeleton is a dynamic and metabolically active tissue, with skeletal stem cells (SSCs) positioned at the top of the differentiation hierarchy of bone cell lineages.^[^ ^2^, ^5^ ^]^ These cells exhibit self‐renewal capacity and multipotent differentiation potential. The skeletal stem cell system comprises a complex lineage network involving mesenchymal stem cells (MSCs) and hematopoietic stem cells (HSCs), which collectively maintain bone and bone marrow homeostasis.^[^ ^6^ ^]^ Both MSCs and HSCs are highly heterogeneous populations, consisting of distinct functional subgroups. Aging of the skeletal system is closely linked to senescence and altered heterogeneity in MSCs and HSCs.^[^ ^7^ ^]^ With advancing age, bone deterioration occurs due to a decline in bone‐forming cells (osteoblasts), which is associated with shifts in MSC differentiation potential and increased osteoclast formation.^[^ ^2^, ^8^ ^]^ Concurrently, HSCs exhibit skewed differentiation toward myeloid lineages. During aging, HSCs accumulate mitochondrial reactive oxygen species (ROS), leading to genotoxic stress and DNA damage.^[^ ^9^ ^]^

HSCs and MSCs possess self‐renewal and multidirectional differentiation potential, enabling tissue renewal and repair as mature cells age. However, stem cells themselves undergo aging.^[^ ^6^, ^7^, ^10^ ^]^ With advancing age, their self‐renewal and differentiation capacities decline due to DNA damage, oxidative stress, and microenvironmental changes.^[^ ^11^ ^]^ HSC numbers and functionality decrease markedly during aging; elderly individuals exhibit a >50% reduction in HSCs compared to younger populations,^[^ ^12^ ^]^ driven by cumulative division and differentiation.^[^ ^6^, ^13^ ^]^ In diseases or injuries requiring extensive regeneration, HSC depletion can lead to exhaustion.^[^ ^2^, ^12^ ^]^ Similarly, MSCs—a heterogeneous population capable of differentiating into chondrocytes, osteoblasts, and adipocytes^[^ ^14^ ^]^—lose migration ability, multipotency, and growth factor production upon senescence. At the cellular level, tissue aging arises from shifts in cellular identity and heterogeneity.^[^ ^2^, ^10^, ^15^ ^]^ Thus, understanding skeletal system aging requires identifying age‐specific skeletal stem cell (SSC) subgroups with distinct functional properties.

While in vitro cell culture is commonly used to characterize stem cells, it is important to note that target cells may not fully replicate their native biological behavior due to significant differences between culture conditions and in vivo environments. Advances in sequencing technology and the rise of precision medicine have enabled powerful tools for studying SSCs across age groups.^[^ ^16^ ^]^ Among these, single‐cell transcriptome sequencing (scRNA‐seq) stands out as an essential method for identifying and labeling rare cell populations, as well as inferring their dynamic changes. This makes scRNA‐seq particularly valuable for research on age‐related osteoporosis.

In our recent research, we discovered that Ti with a micro‐nano surface morphology (MNT) exhibits a paradoxical balance between osteogenesis and senescence. While MNT enhances osteointegration, it also accelerates senescence in bone mesenchymal stem cells (BMSCs).^[^ ^17^ ^]^ Building on these findings, this study aims to design a titanium implant tailored to address the unique pathophysiology of senile osteoporosis. To achieve this, we screened and analyzed single‐cell transcriptome data from SSCs isolated from rat bone marrow of varying ages. This approach enabled identification of specific cell subgroups, their senescence patterns, and differentiation trajectories. Cell experiments elucidated the relationship between BMSC lineage alterations and the progression of age‐related osteoporosis, revealing gene expression changes and key regulatory pathways at distinct time points. To validate these findings, we obtained transcriptome sequencing data from plasma samples of senile osteoporosis patients via a human gene database (e.g., Gene Expression Omnibus (GEO)). Bioinformatics analysis identified underlying mechanisms and critical genes associated with the disease. Based on these insights, we screened out resveratrol (Res)—a compound targeting key genes in senile osteoporosis—for further investigation.

**Scheme 1:**
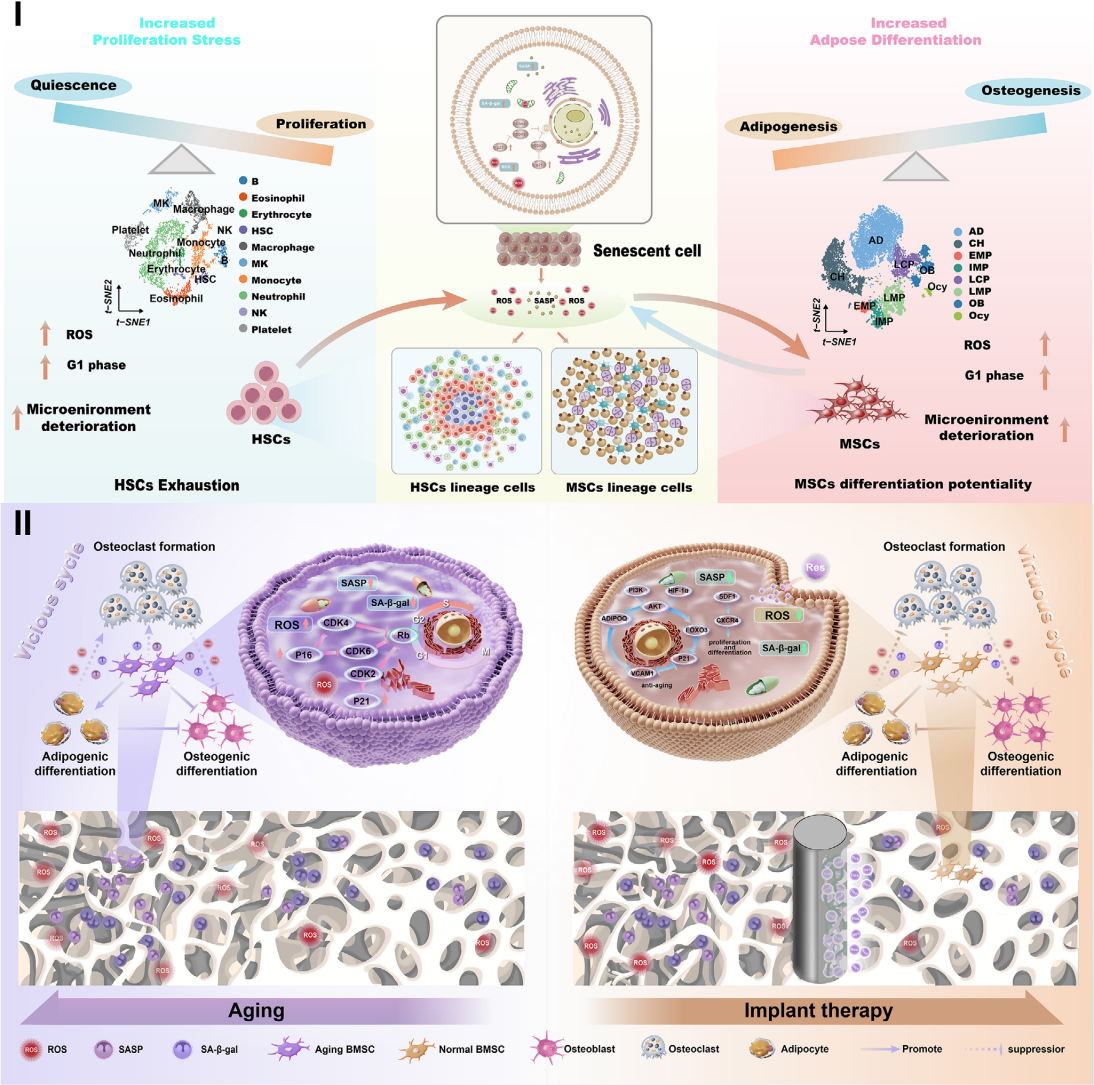
Schematic representation. (I) The dynamics of the skeletal stem cell and (II) its application to the design of a titanium implant for the treatment of senile osteoporosis.

Based on scRNA‐seq and plasma RNA‐seq results, we developed a titanium implant with a ROS ‐responsive hydrogel coating in this study (**Scheme** **1**). In the pathological microenvironment of age‐related osteoporosis, characterized by elevated ROS levels, the thioketal bonds in the hydrogel coating are cleaved, enabling controlled release of Res. This scavenges ROS accumulated in senescent adipocytes, BMSCs, and the surrounding microenvironment, thereby inhibiting secretion of senescence‐associated secretory phenotype (SASP) factors. This shifts the microenvironment around the implant from a “vicious cycle” to a “virtuous cycle”. The synergistic action of Res and the modified Ti surface enhances osteogenic differentiation of aged BMSCs while suppressing osteoclast formation, improving bone‐implant integration in age‐related osteoporosis. To elucidate the underlying mechanisms, we analyzed the implant's effects at RNA, protein, and tissue levels. This study employs scRNA‐seq technology to facilitate the labeling of rare cell populations within bone stem cells, thereby enabling the inference of their differentiation processes. The combination of transcriptome sequencing methods with other techniques provides a foundation for advancing therapeutic strategies and bone repair materials for age‐related osteoporosis.

## 2 Results

### 2.1 A Single‐Cell Transcriptomic Analysis of Skeletal Stem Cells in Bone Marrow

**Figure 1:**
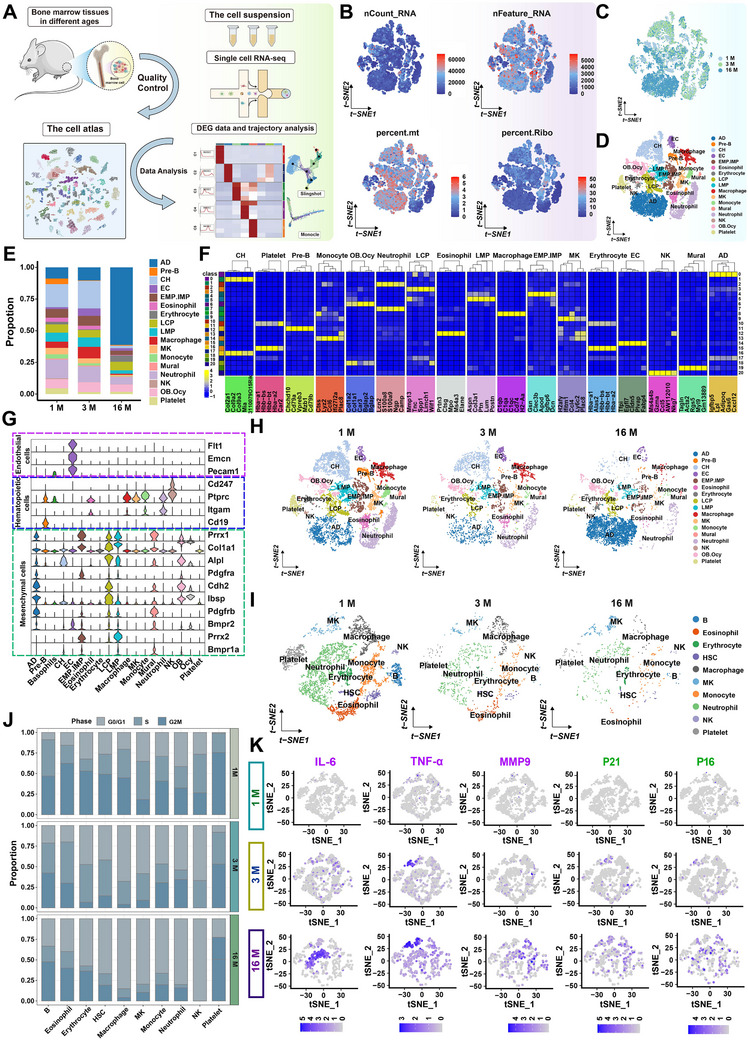
Single‐cell analysis results. A) Illustrations of single‐cell analysis. B) Quality Control: Threshold screening of gene expression, mitochondrial, and ribosomal gene content in samples. C) Cell distribution in 1, 3, and 16 months aged datasets. D) tSNE plots are used to visualize cell types. E) Cell distribution in different age groups. F) Marker genes of different cell subsets and their average expression levels. G) Specific markers for endothelial cell, bone marrow mesenchymal, and hematopoietic cell lineages. H) Annotation on different cell subpopulations at 1, 3, and 16 months. I) Annotation of cell subpopulations in hemopoietic lineages cell at 1, 3, and 16 months. J) Cell cycles of hematopoietic lineage cells from 1, 3, and 16 months. K) Expression of senescence‐related secretory phenotypes (*IL‐6*, *TNF‐α*, and *MMP*) and aging markers (*p21* and *p16*) in hematopoietic lineage cells.

To map the single‐cell transcriptional profile of skeletal stem cells (SSCs) in bone marrow, original bone marrow single‐cell data at 1, 3, and 16 months were obtained from the GEO database (GEO Series: GSE145477) (**Figure** **1**A; Figure S1A, Supporting Information). The selected features, namely RNA (200 to 6000), nCount_RNA (200 to 2000), percent_mito (0.0% to 5.0%), and percent_ribo cells (0.0% to 5.0%), were subjected to tracking and subsequent analysis. Following quality control, a total of 23759 cells were subjected to analysis (Figure 1B). The cells were subjected to dimensionality reduction to integrate the data and were subsequently plotted on the tSNE graph (Figure 1B). It was evident that the bone marrow cells from disparate age groups exhibited a consistent clustering pattern, as illustrated in Figure 1C. The cluster analysis of the gene expression profiles was conducted using Seurat V2, resulting in the identification of 17 cell clusters. Among these, 8 were of the mesenchymal lineage of the bone marrow and 9 were of the hematopoietic lineage (Figure 1D; Figure S1B, Supporting Information). The full names and abbreviations of these cell clusters are listed in the Table S1 (Supporting Information) for reference. The proportions of bone marrow‐derived cell types at 1, 3, and 16 months are presented as a bar graph (Figure 1E). The number of neutrophils, macrophages, and monocytes within the hematopoietic lineage cells decreased considerably at the 16‐month mark. Conversely, the proportion of adipocytes within the mesenchymal lineage cells increased significantly over the same period. The percentage of chondrocytes (CH) exhibited a rapid decline, while the proportions of osteoblasts (OB) and osteocytes (Ocy) remained unaltered.

To investigate the potential causes of the observed alterations in cellular proportions, the expression levels of highly variable genes in each cell subgroup were examined and presented in Figure 1F. It was determined that distinct cell subgroups exhibited differential gene expression. For instance, Lcn2, S100a8, S100a9, Ngp, and Camp were uniquely expressed in neutrophils, whereas Tfpi, Egfl7, Cldn5, Plvap, and Fabp4 were exclusively expressed in endothelial cells. Subsequently, endothelial cells, hematopoietic cell lineages, and bone marrow mesenchymal lineages were identified based on specific gene expression patterns observed in different cell subsets. As illustrated in Figure 1G, the endothelial cells exhibited high expression of Flt1, Ecmcn, and Pecam, while the hematopoietic cell lineages demonstrated high expression of Cd247, Ptprc, Itgam, and Cd19. Col1a1, Alpl, Prrx1, and the other 10 genes exhibited high expression levels in bone marrow mesenchymal lineages.

Bone marrow cells from 1, 3, and 16 months of age were extracted and found to exhibit the same annotated subpopulation (Figure 1H). However, with increasing age, a notable discrepancy in the quantity of bone marrow cells is observed. Subsequently, hematopoietic lineage cells and mesenchymal lineage cells were extracted according to the specific expression genes depicted in Figure 1G, and a dimension reduction cluster analysis was performed once more to obtain the subsets of HSCs and MSCs. In the case of hematopoietic lineage cells (Figure 1I; Figure S2, Supporting Information), the number of cells, except megakaryocytes (MK) in the 3‐ and 16‐month groups, exhibited a reduction compared to the 1‐month group. This reduction was particularly evident in the numbers of neutrophils, macrophages, and monocytes.

The cell cycle of hematopoietic lineage cells was subjected to analysis. As illustrated in Figure 1J, the bone marrow neutrophils, macrophages, natural killer (NK) cells, and monocytes exhibited a block in the G0/G1 phase at both the 3‐ and 16‐month time points. After accounting for age‐related variables, we identified senescence‐related secretion phenotypes and aging markers of hematopoietic lineage cells from different time points. As illustrated in Figure 1K, with the progression of time, hematopoietic lineage cells, including MK and neutrophils, were observed to secrete a considerable quantity of SASP factors, such as IL‐6, TNF‐α, and MMP9. Additionally, the senescence‐related markers P16, P21, and proteins belonging to the cyclin‐dependent kinase protein family (CDK2, CDK4, and CDK6) demonstrated alterations over time (Figure S3, Supporting Information). Our findings suggest that with the advancement of age, the hematopoietic stem cells in the rat bone marrow underwent the initial process of aging. The proliferation of hematopoietic stem cells is inhibited and the number of hematopoietic stem lineage cells exhibited a notable decline in conjunction with the elevated secretion of SASP factors by MK and neutrophils.

### 2.2 A Single‐Cell Transcriptomic Analysis of the Dynamics of Bone Marrow Mesenchymal Lineage Cells

**Figure 2:**
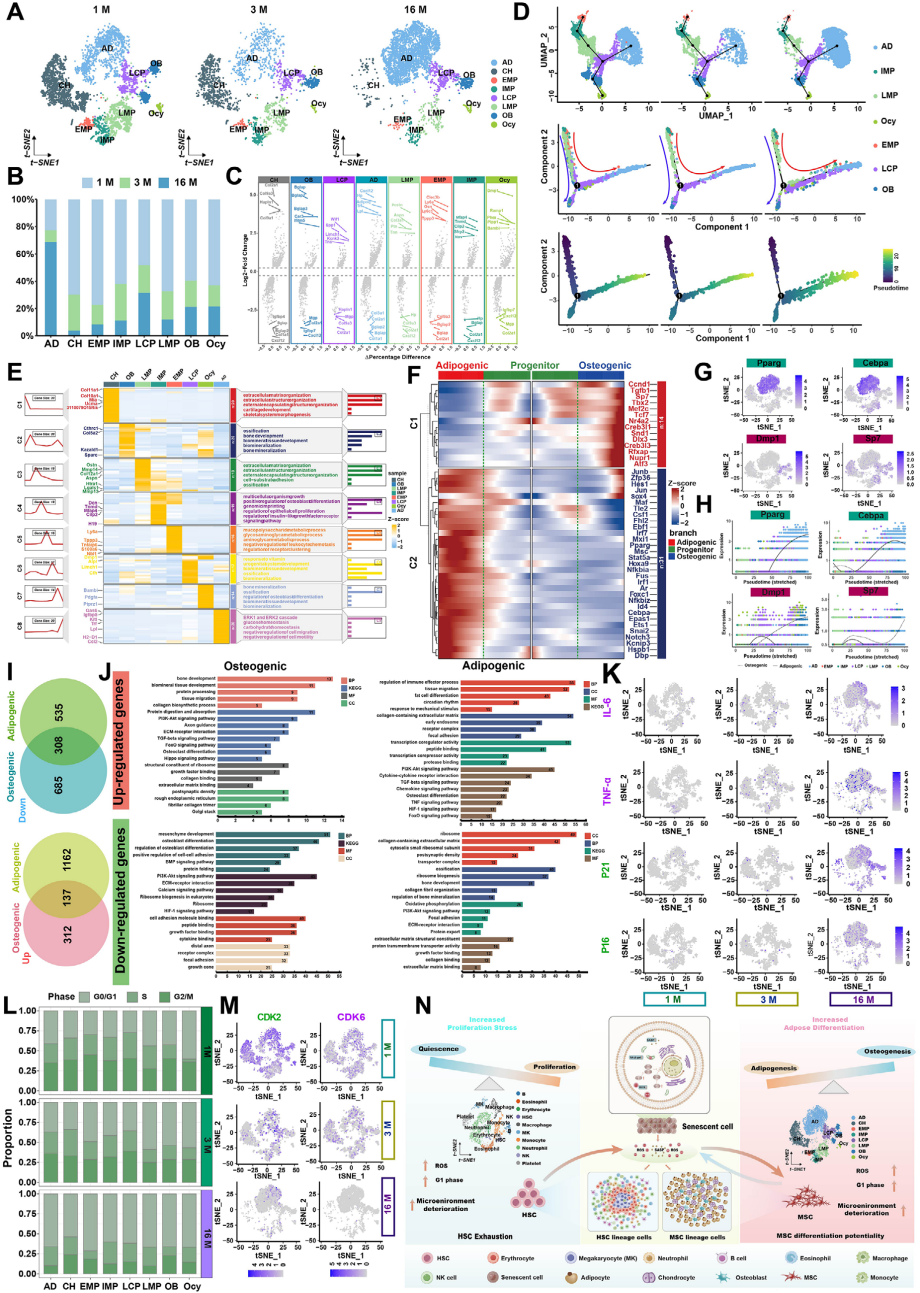
Single‐cell analysis results of bone marrow mesenchymal lineage cells. A) Pseudotime trajectory of bone marrow mesenchymal lineage cells. B) Pseudotime trajectory and functional enrichment of marker genes in bone marrow mesenchymal lineage cells. C) Pseudotime analysis of differentially expressed transcription factors in bone marrow mesenchymal lineage differentiation. D) Expression of transcription factors associated with osteogenic differentiation and lipogenic differentiation. E) Pseudotime trajectory of transcription factors associated with osteogenic differentiation and lipogenic differentiation. F) Number of differential genes during osteogenic and lipogenic differentiation. G) GO and KEGG enrichment analysis during osteogenic and lipogenic differentiation. H) Expression of senescence related secretory phenotypes and aging markers in bone marrow mesenchymal lineages derived from 1, 3, and 16 months of age. I) Cell cycles of bone marrow mesenchymal lineage cells from 1, 3, and 16 months of age.

Mesenchymal lineage cells were extracted according to the specific expression genes, and the subsets of bone marrow mesenchymal lineage cells were obtained (**Figure** **2**A; Figure S4, Supporting Information). The analysis demonstrated that the cell subsets of bone marrow mesenchymal lineage cells from different age groups were identical, including early bone marrow mesenchymal progenitor cells (EMP), intermediate bone marrow mesenchymal progenitor cells (IMP), and late bone marrow mesenchymal progenitor cells (BMP), late mesenchymal progenitor cells (LMP), stereotyped mesenchymal progenitor cells (LCP), osteoblasts (OB), bone cells (Ocy), adipocytes (AD) and chondrocytes (CH). However, the proportion of these cells varies between different age groups. As shown in Figure 2B, the proportion of adipocytes is markedly elevated at 16 months in comparison to 1 and 3 months. Nevertheless, the proportion of other cells is highest at 1 month.

The highly expressed genes among different cell subsets were analyzed for the extracted mesenchymal lineage cell subsets (Figure 2C). The findings demonstrated that distinct cell subsets were regulated by disparate specific expression genes. The selective expression of genes enables the differentiation of mesenchymal progenitor cells in the bone marrow into various cell types.^[^ ^18^ ^]^ Notably, the gene with the highest expression in adipocytes (CXCL12) exhibits the lowest expression in osteoblasts. Conversely, the two most highly expressed genes in osteoblasts (Bglap and Bglap2) demonstrate a significant downregulation in adipocytes. These findings indicate that the differentiation of MSCs into adipocytes and osteoblasts is mutually exclusive.

The alterations in the mesenchymal lineage cells over time were inferred by utilizing the Monocle V2 and Slingshot software packages. The findings indicated that the mesenchymal lineage cells exhibited a consistent differentiation pattern across different age groups. In summary, the differentiation process commenced with the EMP, proceeded through the IMP, LMP, and LCP stages, and ultimately culminated in the differentiation into osteoblasts or adipocytes (Figure 2D; Figure S5A, Supporting Information). It should be noted that the differentiation trajectory between osteoblasts and adipocytes exhibits age‐related variations. Following the LCP stage, the primary differentiation trajectory is toward osteoblasts at one month, while at 16 months it is toward adipocytes. During this process, genes that are specifically expressed in different cell subsets regulate the direction of EMP differentiation and the biological processes that occur during this process (Figure 2E; Figure S5B, Supporting Information).

The pseudotime map of differentially expressed transcription factors, derived from EMP, revealed the transcription factors that regulate osteogenic and adipogenic differentiation (Figure 2F). Several transcription factors have been identified as potential regulators of adipogenic differentiation of EMP, including several known adipogenesis‐related transcription factors (e.g., Pparg, Cebpa, Fus, and Ebf1) and other novel transcription factors.^[^ ^19^ ^]^ Nevertheless, the number of transcription factors involved in the regulation of osteogenic differentiation is smaller, indicating that adipocyte differentiation is more readily achieved than osteoblast differentiation in the absence of external factors. Further analysis was conducted on the expression of key transcription factors associated with osteogenic and adipogenic differentiation (Figure 2F) and their pseudo‐time loci. Among the identified transcription factors, Damp1 and Sp7 are predominantly expressed during osteogenic differentiation, while Pparg and Cebpa are primarily expressed during adipogenic differentiation (Figure 2G,H). Moreover, Pparg and Cebpa show a consistent increase in expression over time, whereas Damp1 and Sp7 exhibit distinct fluctuations in their expression patterns.

The data indicated that there was some overlap in the differentially expressed genes (DEGs), suggesting that they play a pivotal role in both osteogenic and adipogenic differentiation. Functional enrichment analysis of the differentially expressed genes (DEGs) according to the Gene Ontology (GO) and the Kyoto Encyclopedia of Genes and Genomes (KEGG) revealed both unique and common features during osteogenic and adipogenic differentiation (Figure 2J). Some pathways, including the PI3K‐Akt, TGF‐β, and FOXO signaling pathways, are altered during both osteogenic and adipogenic differentiation, indicating that they play a general role in regulating differentiation. The distinctive characteristics of osteogenic differentiation encompass the organization of the extracellular matrix (ECM), ECM‐receptor interactions, axon guidance, actin cytoskeleton, and biomaterial tissue development. These characteristics are indicative of their bone‐forming functions. It is noteworthy that the distinctive characteristics of adipogenic differentiation encompass cytokine‐cytokine receptor interactions, immune system processes, osteoclast differentiation, TNF, HIF‐1, chemokine pathways, and so forth. Given that adipocytes are situated within a vascular‐rich hematopoietic milieu, these observations suggest that hematopoietic stem cells may exert a pivotal regulatory influence over the process of adipogenesis.

The preceding analysis indicates that EMPs with an identical hierarchical differentiation pattern are responsible for the differentiation of mesenchymal lineages at varying stages of development. However, during the process of aging, there is not only a reduction in the number of EMPs but also a tendency for these cells to differentiate toward an adipogenic phenotype. To gain insight into the underlying mechanisms, senescence‐related secretion phenotypes and aging markers of mesenchymal lineage cells from different ages were identified. As illustrated in Figure 2K, the levels of SASP factors and senescence‐related markers in mesenchymal lineage cells exhibited a notable increase with advancing age, particularly at 16 months. It is noteworthy that a considerable quantity of SASP factors, including IL‐6 and TNF‐α, were secreted by adipocytes. However, the senescence‐related markers P16 and P21 were predominantly expressed in LCP and OB. Additionally, cell cycle analysis (Figure 2L) demonstrated that all mesenchymal lineage cells in the bone marrow were arrested in the G0/G1 phase. Meanwhile, the proteins belonging to the cyclin‐dependent kinases protein family (CDK2, CDK4, and CDK6), which are closely related to the cell cycle, were analyzed in Figure 2L and Figure S6 (Supporting Information). This may further explain the observed decrease in activity and differentiation of mesenchymal progenitor cells (Figure 2I).

In light of the evidence presented in both Figures 1 and 2, we posited that SSCs will undergo an aging process as a consequence of increasing age (Figure 2M), with HSCs exhibiting signs of early aging. The secretion of SASP factors by aged neutrophils and MKs would promote the differentiation of MSCs into adipocytes. Concurrently, adipocytes secrete copious quantities of SASP factors, which induce the senescence of LCP and OB in MSCs. Furthermore, the proliferation of all mesenchymal lineage cells is inhibited, as is the osteogenic differentiation of MSCs.

### 2.3 The Bone Marrow Analysis and Molecular Mechanism for Senile Osteoporosis

**Figure 3:**
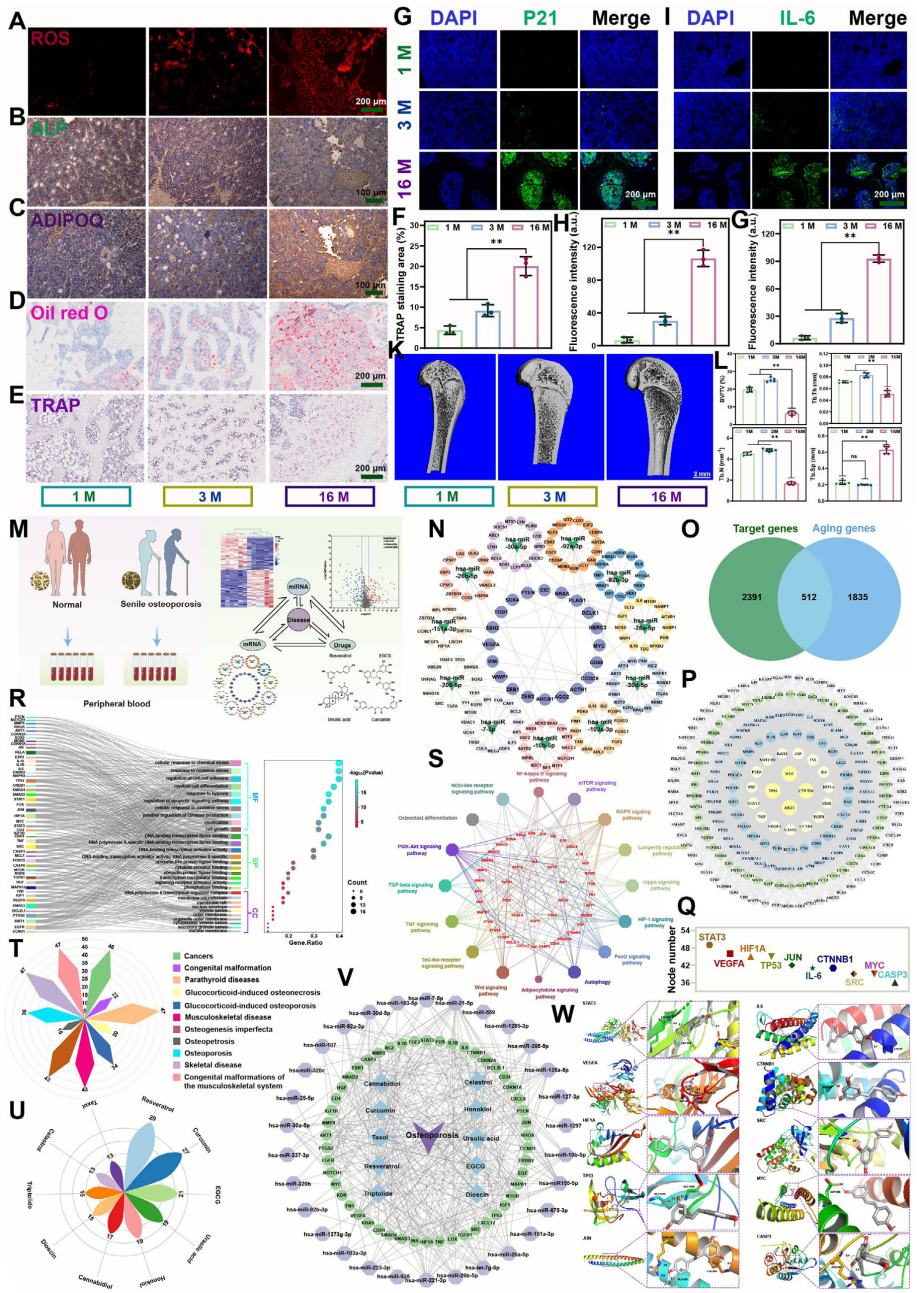
Analysis at the biological level and screening of target drugs. A) Representative images of frozen sections of bone tissue from different ages show ROS fluorescence in different samples. Scale bar: 200 µm. Immunohistochemical staining of ALP B) and ADIPOQ C) of bone tissue samples from different ages. Scale bar: 100 µm. D) Oil red O staining image of bone tissue samples from different ages. Scale bar: 200 µm. TRAP staining images E) and quantitative analysis F) of bone tissue samples from different ages (n = 3). Scale bar: 200 µm. Representative fluorescence images G) and relative fluorescence density H) of p21 in bone tissue samples from different age sources (n = 3). Scale bar: 200 µm. Representative fluorescence images I) and relative fluorescence density J) of IL‐6 in bone tissue samples from different age sources. Scale bar: 200 µm. K) Representative micro‐CT images of the distal femur of rats at different ages. Scale bar: 2 mm. L) Quantitative analysis of new bone volume per total volume (BV/TV), trabecular thickness (Tb. Th), trabecular number (Tb. N) and trabecular bone separation (Tb. Sp) in the bone samples (n = 5). M) Schematic diagram of target drug screening for senile osteoporosis. N) Diagram of the corresponding target gene network of the differentially expressed miRNA. O) Venn diagram showing the targeted and age‐related gene sets. P) Protein‐protein interaction networks of target genes. Q) Number of nodes in the core protein interaction network. R) GO annotation analysis of hub target genes. S) KEGG enrichment analysis of hub target genes. T) Disease analysis of hub target genes U) Results of drug target screening. V) Disease‐drug – miRNA – target interaction network. W) The molecular docking of the hub genes (STAT3, VEGFA, HIF1A, TP53, JUN, IL‐6, CTNNB1, SRC, MYC, and CASP3) and Res. Data were presented as mean values ± standard deviations (SD); error bars = SD. *p < 0.05, **p < 0.01.

Bone marrow was extracted from Sprague‐Dawley rats of varying ages, and the samples were subjected to fluorescence and immunohistochemical staining. As illustrated in **Figure** **3**A–F, the bone marrow of 16‐month‐old rats exhibits the highest expression of ROS, adiponectin (ADIPOQ), Oil red O, and tartrate‐resistant acid phosphatase (TRAP). This indicates that as age increases, the number of reactive oxygen species, adipocytes, and osteoclasts in the bone marrow also increases. Conversely, the expression of alkaline phosphatase (ALP) is the lowest in the bone marrow at 16 months, indicating the lowest number of osteoblasts. Similar results were found in experiments at the cellular level (Figure S6B–G, Supporting Information). Moreover, as age increased, the levels of the aging markers (P21 and IL‐6) in the bone marrow rose gradually (Figure 3G–J). It can be concluded that the 16‐month‐old mice exhibited osteoporosis‐related manifestations, including a reduction in bone mass and alterations in the trabecular structure (Figure 3K,L).

To verify and elucidate the potential pathogenesis of senile osteoporosis from the clinic, miRNA data from the GEO database (GEO Series: GSE93883) were downloaded for analysis (Figure 3M). The differential expression of miRNAs in plasma samples from senile patients with and without osteoporosis was initially examined. A total of 220 differentially expressed miRNAs (│log_2_FC│>2, *P‐value* <0.05) were screened and their relationships were represented by heat and volcano maps as shown in Figure S7 (Supporting Information). The miRNAs with significant differences were selected for the construction of target gene networks, which revealed the existence of both common and unique target genes corresponding to miRNAs with disparate differences (Figure 3N) The aging process is associated with an increased risk of developing osteoporosis. In light of the pathological characteristics observed in clinical manifestations, a search was conducted for 512 overlapping genes between the target gene set and the aging gene set, as illustrated in the Venn diagram in Figure 3O. The protein‐protein interaction network (PPI) and Matthews correlation coefficient (MCC) algorithms were employed to analyze 512 overlapping and hub genes to elucidate the significance of key genes in the regulation of senile osteoporosis. As illustrated in Figure 3P, the 50 pivotal genes encompass STAT3, VEGFA, HIF‐1A, TP53, JUN, MYC, CTNNB1, IL‐6, SRC, and CASP3 (Figure 3Q), and others. These key genes are intimately linked with the processes of aging, oxidative stress, and bone metabolism. The results of the Gene Ontology (GO) enrichment analysis (Figure 3R) indicated that the functions of the top 50 genes were primarily associated with biological processes such as chemical stress response, oxidative stress response, bone development, bone mineralization, ossification, cell growth and differentiation, and cell cycle. The KEGG enrichment results (Figure 3S) demonstrated that the top target genes predominantly regulated the PI3K‐Akt signaling pathway, osteoclast differentiation, the mTOR signaling pathway, the longevity regulation pathway, the HIF‐1 signaling pathway, the FOXO signaling pathway, autophagy, the TNF signaling pathway, and so forth. Subsequently, a disease correlation analysis revealed that the diseases associated with these core target genes were concentrated in the musculoskeletal system, including skeletal diseases, musculoskeletal disorders, congenital malformations of the musculoskeletal system, osteosclerosis, osteogenic dysplasia, and so forth (Figure 3T). These findings indicate that several genes may be involved in the regulation of senile osteoporosis, which adds another layer of complexity to the management of this disease.

Based on the literature analysis and the HERB database, the molecular drugs corresponding to 50 selected core genes and their related components were predicted and analyzed. A total of 326 related drug components were identified to be closely related to the core target genes, among which resveratrol (Res) had the highest correlation with the core target genes (Figure 3U). Furthermore, the integration of differential miRNA, core target genes, molecular drugs, and disease interaction networks provided additional insight into the target, mechanism, physiological, and pathological processes of drug action (Figure 3V). Subsequently, to corroborate the correlation between the selected drugs and the core genes, the interaction between resveratrol (Res) and the active sites in the top ten core genes was investigated by molecular docking experiments. These experiments were conducted following the principles of spatial structure complementarity and energy minimization. The optimal binding position and strength of Res with these genes were successfully determined (Figure 3W).

### 2.4 Preparation and Characterization of Res@TNT‐PDA/Gel

**Figure 4:**
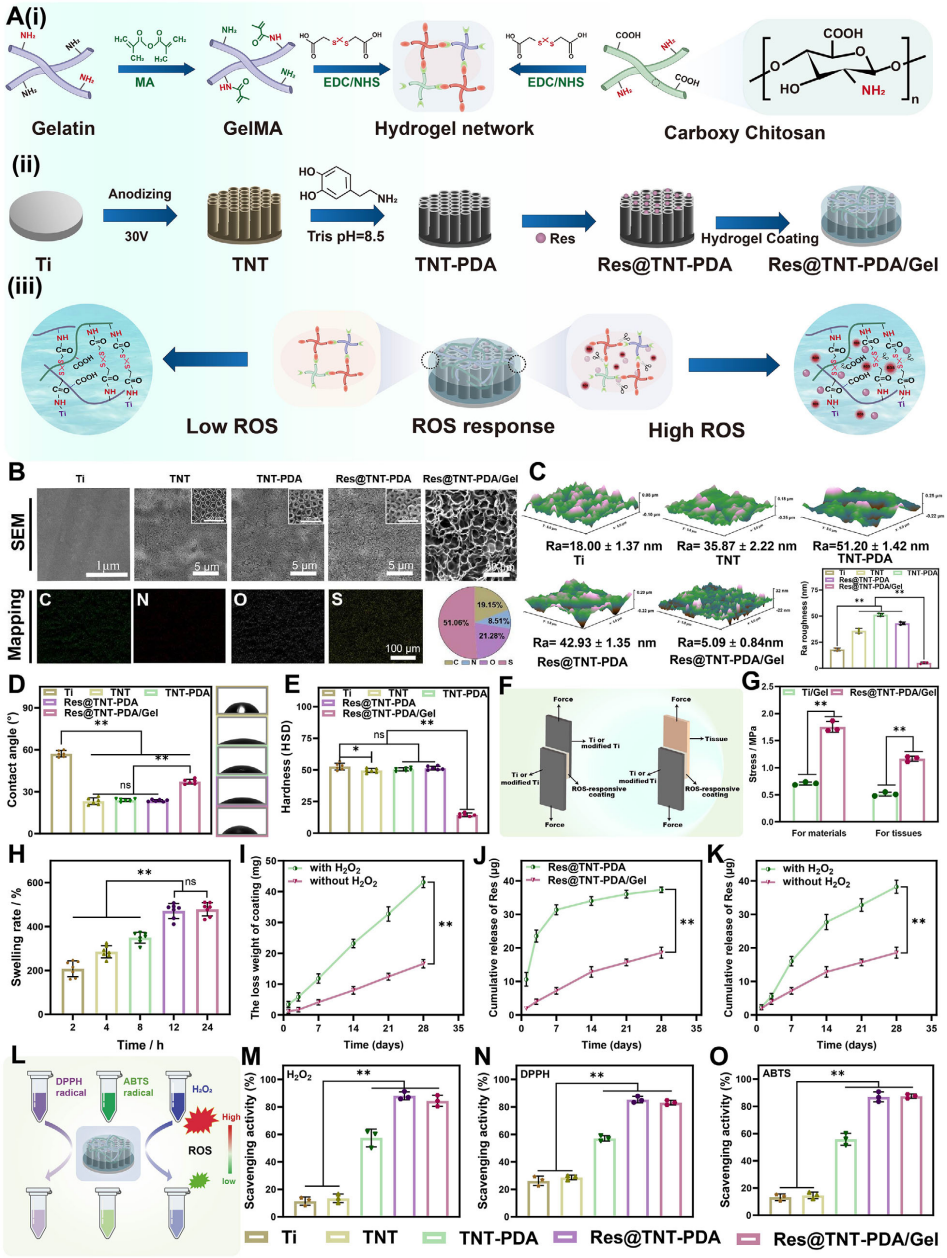
Structural and functional characterizations of the modified Ti. A‐i) Synthesis procedure of hydrogel. A‐ii) Synthesis diagram of the Ti surface modification (Res@TNT‐PDA/Gel). A‐iii) Schematic diagram of ROS‐responsive hydrogel coating degradation. B) SEM images of different Ti substrates and mapping images and atomic ratio of Res@TNT‐PDA/Gel. C) AFM images of different Ti substrates and quantitative statistical results of roughness (n = 3). D) Contact angles of various Ti surfaces (n = 6). E) Hardness of various Ti surfaces (n = 6). F) Schematic illustration of the bond strength measurement. G) Average adhesion strength of the ROS‐responsive hydrogel coating (n = 3). H) Swelling rate of ROS‐responsive hydrogel coating (n = 7). I) The weight loss of ROS‐responsive coating when incubating with PBS with or without H_2_O_2_ (n = 3). J) Release profiles of Res from Res@TNT‐PDA and Res@TNT‐PDA/Gel samples (n = 3). K) Release profiles of Res when incubating with PBS with or without H_2_O_2_ (n = 3). L) Schematic diagram of the antioxidant test. M) H_2_O_2_ scavenging capacity of different samples (n = 3). N) DPPH radicals scavenging capacity of different samples (n = 3). O) ROS scavenging capacity of different samples (n = 3). Data were presented as mean values ± standard deviations (SD); error bars = SD. ns>0.05, *p < 0.05, **p < 0.01.

To enhance the capacity of the implant to integrate with the surrounding bone tissue in patients with senile osteoporosis, a novel implant was developed in this work which is capable of reversing the differentiation of aged BMSCs into adipogenesis. This has the potential to alleviate the effects of aging and reduce oxidative stress around the bone tissue. An anodic oxidation technology was employed to prepare titanium dioxide nanotube‐like structures on the surface of titanium implants for the loading of Res. Subsequently, a ROS‐responsive hydrogel coating was constructed on the surface of the modified titanium, comprising a two‐group hydrogel network containing methacrylate‐modified gelatin and carboxylated chitosan (**Figure** **4**A; Figure S8A, Supporting Information).

GelMA is a precursor for the preparation of ROS‐responsive hydrogel coatings, which are formed by the reaction of methacrylate anhydride with an amino group on gelatin. The results of the 1H NMR analysis indicated the presence of a chemical shift peak between 5 and 6 ppm, which was attributed to the two hydrogen protons of the alkenyl group situated above the double bond of the grafted methacrylate (Figure S8B, Supporting Information). Furthermore, the FTIR spectra revealed that the GelMA peaks were more pronounced at 1634 and 3284 cm^−1^ in comparison to gelatin (Figure S8C, Supporting Information). The results demonstrated the successful synthesis of the GelMA polymer. The alterations in surface morphology throughout the preparation process were examined using scanning electron microscopy (SEM), as illustrated in Figure 4B. The results demonstrate that the TNT structure has a diameter of ≈110 nm and a thickness of ≈1 µm (Figure S9A, Supporting Information). The arrangement of the TNT structure was found to be ordered, and the introduction of the polydopamine layer and Res did not result in a significant change to the topology of the modified titanium. The ROS‐responsive hydrogel coating allows for the construction of a uniform porous network structure around the modified titanium, which effectively shields the underlying topology. The EDS spectrum analysis results demonstrate the presence of the S element on the Res@TNT‐PDA/gel surface, with an S element content of 51.06%. The AFM test results indicate that the arithmetic mean roughness (Ra) of each group was 18.00 ± 1.37 nm, 35.87 ± 2.22 nm, 51.20 ± 1.42 nm, 42.93 ± 1.35 nm, and 5.09 ± 0.84 nm, respectively (Figure 4C). It was observed that the surface roughness of the TNT, TNT‐PDA, and Res@TNT‐PDA groups exhibited a notable increase in comparison to that of the pure Ti group throughout the surface modification process of the implants. In contrast to conventional methods of modifying the titanium surface, the construction of the hydrogel coating can impart entirely distinct chemical and physical properties to the titanium surface.^[^ ^20^ ^]^

So far, the porous network structure of the hydrogel coating can not only act as a natural barrier for the underlying drug reservoir and control the rate of drug release, but also its highly hydrophilic and conformable structure can fill the gaps around the implant and bone tissue. The results of the water contact angle test (Figure 4D) show that the average water contact angle on the Res@TNT‐PDA/gel surface is 37.15°, which is 19.98° lower than that on the pure Ti surface. This can be attributed to the presence of abundant hydrophilic groups (such as amino and carboxyl groups) in methacrylate‐modified gelatin and carboxylated chitosan. At the same time, the hardness measurement results (Figure 4E) showed that the hardness of Res@TNT‐PDA/Gel was significantly reduced, which was conducive to promoting cell adhesion and improving the integration effect of the titanium implant with the soft tissue surrounding the bone. Moreover, the hydrogel coating exhibits a robust affinity for modified titanium through an amide reaction. To evaluate the adhesion strength between the hydrogel coating on the titanium implants and the surrounding tissues, a lap shear experiment was conducted (Figure 4F). The results indicate that the adhesion strength of Res@TNT‐PDA/Gel to both materials and tissues is superior to that of pure titanium, with average adhesion strengths of ≈1.75 and 1.16 MPa, respectively (Figure 4G).

The swelling measurement results of Res@TNT‐PDA/Gel demonstrate that following a 24‐hour incubation period, the swelling rate of the hydrogel coating reaches ≈478% (Figure 4H). To examine the ROS response of the hydrogel coating, the Res@TNT‐PDA/gel was submerged in PBS buffer with and without H₂O₂, and the extent of loss was quantified at designated intervals. As illustrated in Figure 4I, the degree of solid loss exhibited by the sample increased progressively with the duration of incubation. Furthermore, samples immersed in PBS buffer containing H₂O₂ exhibited accelerated degradation. These findings illustrate that the degradation rate of hydrogel coatings can be expedited by the degradation of 2,2′‐[propane‐2,2‐diylbis (thio)] diacetic acid‐mediated cross‐linking in environments with elevated ROS levels.

The release behavior of resveratrol was investigated utilizing Res@TNT‐PDA and Res@TNT‐PDA/Gel samples. As illustrated in Figure 4J, the release of resveratrol from Res@TNT‐PDA exhibits a bursting phenomenon in comparison to Res@TNT‐PDA/Gel. Subsequently, the release of resveratrol from the Res@TNT‐PDA/Gel samples was monitored in PBS buffer solutions with and without hydrogen peroxide. The findings demonstrated that the release of resveratrol was accelerated with the progressive degradation of the ROS‐responsive hydrogel coatings in an environment with elevated ROS concentrations (Figure 4K). The results demonstrate that the hydrogel coating, as designed, has the effect of controlled drug release, thereby facilitating the maximum application of Res. Moreover, given that senile osteoporosis arises in an environment characterized by elevated levels of ROS, the antioxidant properties of these modified titanium implants were evaluated and demonstrated (Figure 4L–O). The antioxidant effect of the pure Ti and TNT groups was found to be negligible when H₂O₂, DPPH, and ABTS were measured. The TNT‐PDA has been demonstrated to possess antioxidant capacity, which can be attributed to the antioxidant capacity of the PDA. Moreover, the antioxidant activity of Res@TNT‐PDA and Res@TNT‐PDA/gel was demonstrated to be markedly effective against H₂O₂, DPPH, and ABTS.

### 2.5 Cell Activity and In Vitro Anti‐Aging Evaluation

**Figure 5:**
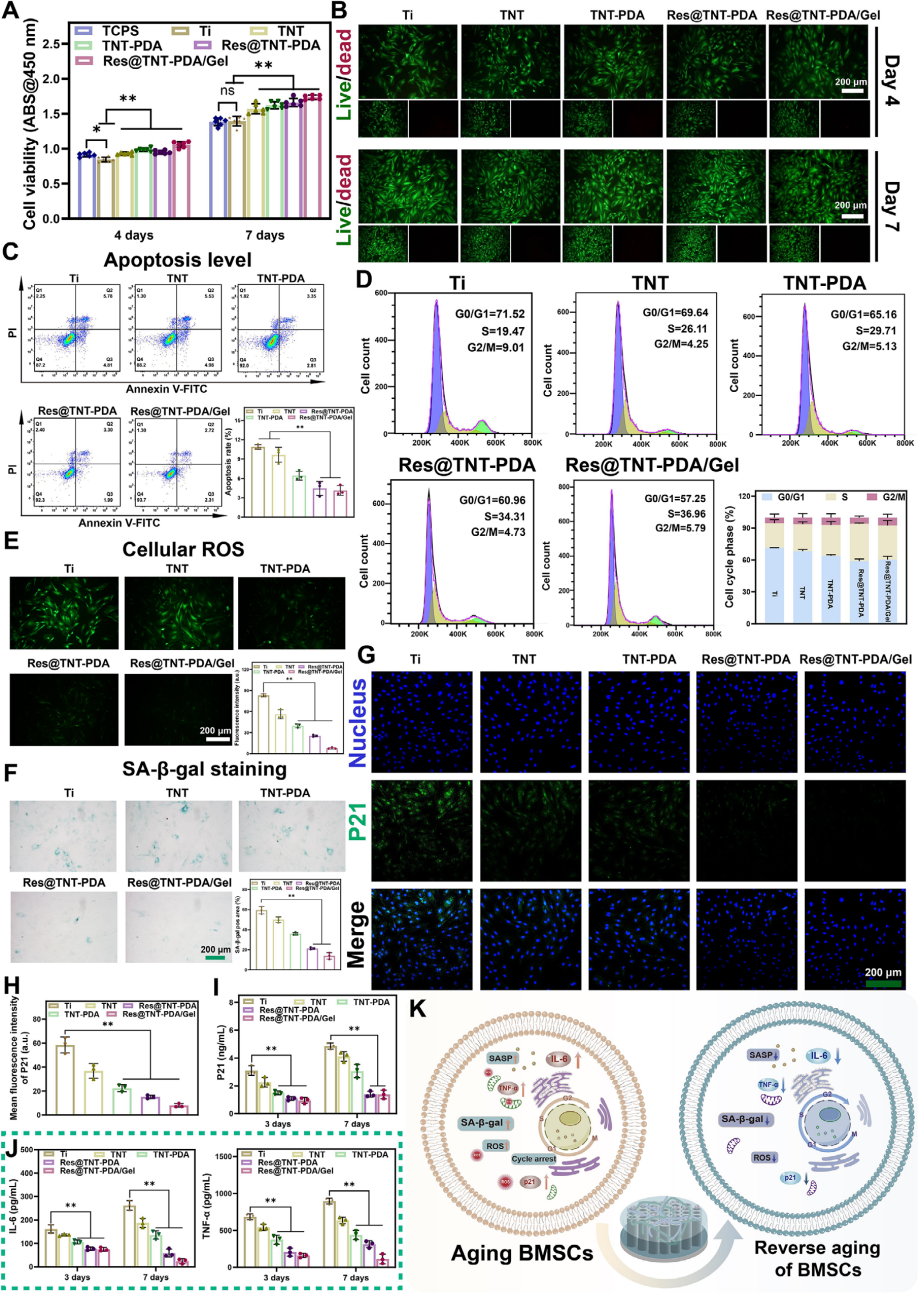
Evaluation of biocompatibility and anti‐aging effect of modified Ti. A) Viability of senescent BMSCs cultured on different samples for 4 and 7 days (n = 6). B) Lived/dead staining of senescent BMSCs on Ti, TNT, TNT‐PDA, Res@TNT‐PDA, and Res@TNT‐PDA/Gel was observed after 4 and 7 days. Scale bar: 200 µm. C) Apoptotic analysis and corresponding quantitative statistics of senescent BMSCs after incubation with different samples for 3 days (n = 3). D) Cell cycle analysis and corresponding quantitative statistics of senescent BMSCs after incubation with different samples for 3 days (n = 3). E) Representative images and corresponding quantitative statistics of senescent BMSCs cellular ROS after incubation with different samples for 3 days (n = 3). Scale bar: 200 µm. F) Representative images and corresponding quantitative statistics of SA‐β‐gal staining after incubation with different samples for 3 days (n = 3). Scale bar: 200 µm. Representative fluorescence images G) and relative fluorescence density H) of p21 of senescent BMSCs after incubated with different samples for 3 days (n = 3). Concentrations of P21 I), and SASP‐related protein J) were secreted by senescent BMSCs after 3 and 7 days of co‐culture (n = 3). K) Schematic diagram of the anti‐aging effect of materials. Data were presented as mean values ± standard deviations (SD); error bars = SD. *p < 0.05, **p < 0.01.

The biocompatibility of the materials was evaluated using CCK‐8 and FDA/PI staining. Additionally, aged BMSCs were separately incubated with the samples for four and seven days. The results demonstrated that the cell viability of the TNT, TNT‐PDA, Res@TNT‐PDA, and Res@TNT‐PDA/gel groups was enhanced in comparison to the pure Ti group (**Figure** **5**A). The FDA/PI staining revealed the presence of only a few dead cells on all surfaces (Figure 5B; Figure S9B, Supporting Information). The results demonstrated that the modified titanium samples exhibited excellent biocompatibility. The level of apoptosis of aged BMSCs on the modified titanium surface was determined by flow cytometry. As illustrated in Figure 5C, the apoptosis rates induced by the Ti, TNT, TNT‐PDA, Res@TNT‐PDA, and Res@TNT‐PDA/gel groups were 10.59%, 10.51%, 6.16%, 5.29%, and 5.03%, respectively. It can be concluded that the Res@TNT‐PDA/Gel group can markedly enhance the viability of aged BMSCs and diminish the incidence of cell apoptosis. Furthermore, to ascertain the impact of modified titanium on the cell cycle of aged BMSCs, the proportional distribution of G0/G1, S, and G2/M phases was calculated by detecting the DNA content (Figure 5D). Following co‐culture, the number of cells in the G0/G1 phase significantly decreased in the Res@TNT‐PDA and Res@TNT‐PDA/Gel groups, while the number of cells in the S phase and G2/M phase increased. This suggests that the Res@TNT‐PDA and Res@TNT‐PDA/Gel groups could alleviate the division barrier of aged BMSCs and promote cell proliferation.

To investigate the scavenging effect of modified titanium on intracellular ROS of aged BMSCs, the cells were detected utilizing a DCFH‐DA probe. The findings indicated that the TNT‐PDA, Res@TNT‐PDA, and Res@TNT‐PDA/gel groups demonstrated a notable ROS scavenging effect in comparison to Ti and TNT. A similar trend was observed in the quantitative analysis of the fluorescence intensity of ROS (Figure 5E). Moreover, the SA‐β‐gal activity assay revealed that Res@TNT‐PDA/gel markedly diminished the SA‐β‐galactosidase‐positive region (Figure 5F). These findings suggest that the modified titanium samples may have the potential to decelerate the aging process of aged BMSCs. Concurrently, the anti‐aging capacity was assessed by quantifying the relative fluorescence intensity of P21. As illustrated in Figure 5G,H, the fluorescence intensity was markedly diminished in the Res@TNT‐PDA and Res@TNT‐PDA/gel groups in comparison to the Ti, TNT, and TNT‐PDA groups. Moreover, the secretion level of P21 demonstrated that Res@TNT‐PDA/Gel was capable of significantly suppressing the expression of P21 (Figure 5I). Prior analyses have demonstrated that senescent BMSCs elicit the secretion of a greater quantity of SASP. Subsequently, the modified titanium was incubated with senescent BMSCs to detect the secretion of SASP. The findings demonstrated that the secretion levels of SASP (IL‐6 and TNF‐α) would progressively increase with an extended co‐culture duration. However, the Res@TNT‐PDA/Gel exhibited the most pronounced inhibitory impact in comparison to the other groups (Figure 5J). In conclusion, the Res@TNT‐PDA/Gel has an excellent safety profile, capable of eliminating the intracellular ROS of senescent cells and inhibiting the secretion level of SASP. Concurrently, Res@TNT‐PDA/Gel is capable of effectively mitigating the senescent state of senescent BMSCs, facilitating cell proliferation, and modulating the senescent microenvironment (Figure 5K).

### 2.6 In Vitro Differentiation and Bone Integration Assessment of BMSCs

**Figure 6:**
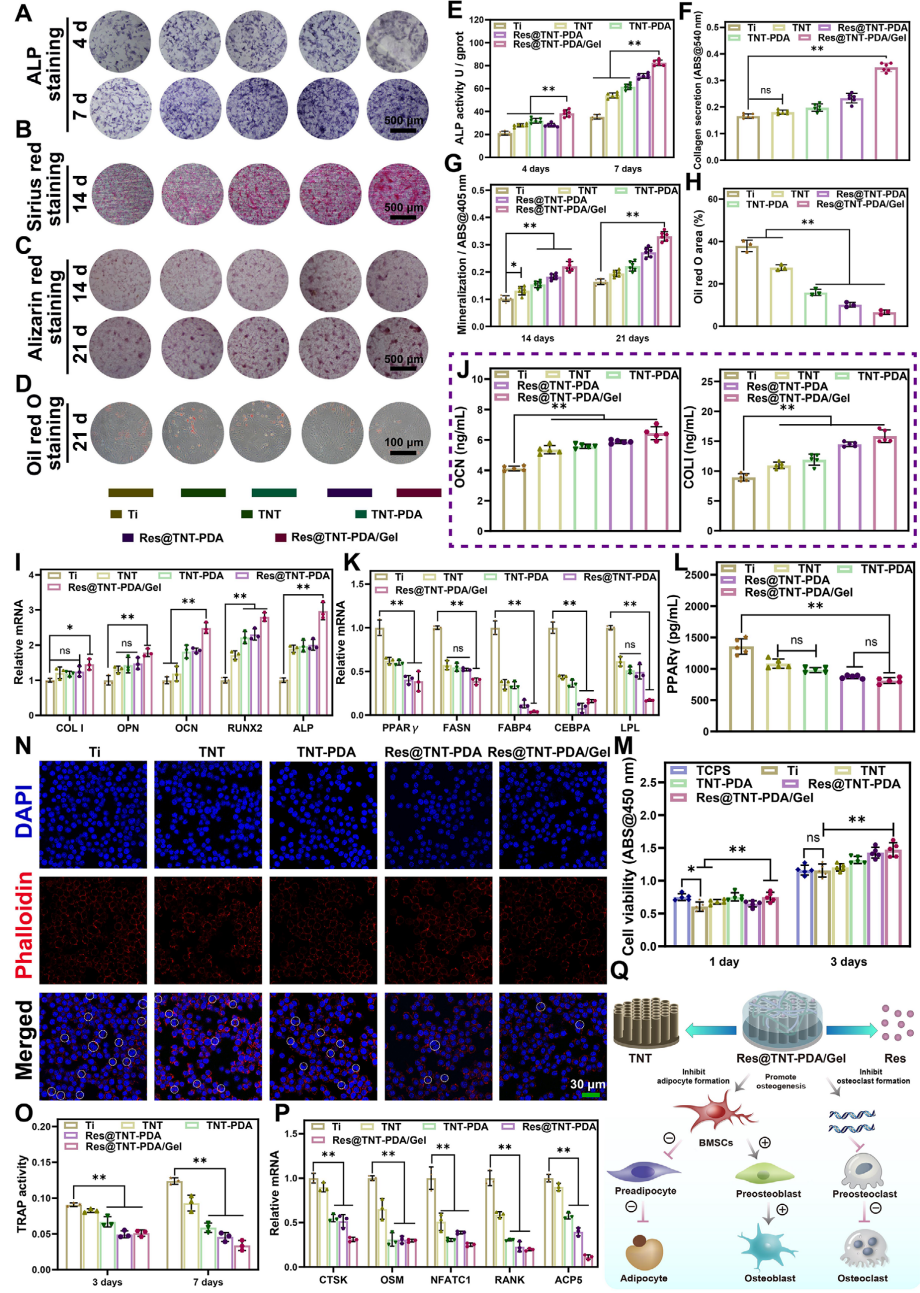
Evaluation of modified Ti to regulate differentiation potential of aging BMSCs and inhibit osteoclastic differentiation. ALP staining A), Sirius red staining B), Alizarin red staining C), and Oil red O staining D) of senescent BMSCs. ALP quantitative E) (n = 6), Collagen quantitative F) (n = 6), and Mineralization quantitative G) (n = 6) and Oil red O quantitative H) (n = 3) analysis of senescent BMSCs. I) Relative mRNA expression of osteogenesis‐ related genes in senescent BMSCs treated by various samples for 7 days (n = 3). J) Concentrations of osteogenesis‐related protein were secreted by senescent BMSCs after 7 days of co‐culture (n = 5). K) Relative mRNA expression of adipogenesis‐related genes in senescent BMSCs treated by various samples for 7 days (n = 3). L) Concentrations of adipogenesis‐related protein were secreted by senescent BMSCs after 7 days of co‐culture (n = 5). M) Viability of Raw264.7 cells cultured on different samples for 1 and 3 days (n = 5). N) Representative images of multinucleated cells on different substrates after culturing for 5 days. Scale bar: 30 µm O) Production of TRAP from RAW264.7 cells on different substrates after 3 and 7 days of incubation (n = 3). P) Relative mRNA expression of osteoclast differentiation‐related genes (n = 3). Q) Schematic diagram of material regulated differentiation. Data were presented as mean values ± standard deviations (SD); error bars = SD. *p <0.05, **p < 0.01.

To evaluate the in vitro osteogenic and adipogenic effects of Ti, TNT, TNT‐PDA, Res@TNT‐PDA, and Res@TNT‐PDA/Gel, a qualitative and quantitative analysis was conducted on ALP activity, collagen secretion, ECM mineralization, and lipid levels (**Figure** **6**A–H). The results of ALP staining (Figure 6A) demonstrated that Res@TNT‐PDA/Gel exhibited the highest ALP production, while the quantitative analysis (Figure 6E) revealed that the ALP activity in the Res@TNT‐PDA/Gel group was markedly elevated in comparison to the other groups. Subsequently, collagen secretion was stained and quantified after 14 days. The results demonstrated that aged BMSCs on the Res@TNT‐PDA/Gel surface exhibited the highest collagen secretion (Figure 6B,F). The degree of extracellular matrix (ECM) mineralization in aged BMSCs was evaluated by alizarin red staining (Figure 6C) and quantitative analysis (Figure 6G), which exhibited a similar trend to that observed for alkaline phosphatase (ALP) and collagen. Furthermore, the expression of osteogenic differentiation genes (COLI, OPN, OCN, RUNX2, and ALP) and adipogenic differentiation genes (PPARγ, FASN, FABP4, CEBPA, and LPL) was analyzed (Figure 6I,K). The Res@TNT‐PDA/Gel has been demonstrated to facilitate the differentiation of aged BMSCs into osteoblasts while simultaneously inhibiting their differentiation into adipocytes. The protein level results corroborate this conclusion (Figure 6J,L).

It is of paramount importance to maintain a dynamic equilibrium between osteoblasts and osteoclasts to effectively treat senile osteoporosis. The modified titanium was co‐cultured with Raw264.7, and the results of the activity test demonstrated that the different material groups exhibited no discernible toxicity to the cells (Figure 6M). Subsequently, the modified titanium was co‐cultured with osteoclasts that had been induced by specific culture conditions. The confocal laser scanning microscopy (CLSM) results demonstrated a notable reduction in the number of multinucleated osteoclasts (marked with white dotted circles) on the surface of Res@TNT‐PDA/Gel (Figure 6N). Additionally, the TRAP activity (a marker of multinucleated osteoclasts) was markedly diminished in the Res@TNT‐PDA/Gel group, particularly on day 7 (Figure 6O). Moreover, the expression levels of the characteristic functional genes of osteoclasts (CTSK, OSM, NFATC1, RANK, and ACP5) were quantified by qRT‐PCR, and a significant decrease in the expression levels of these genes was observed in the Res@TNT‐PDA/Gel group (Figure 6P). These findings provide compelling evidence that the fabricated functional titanium implants can effectively inhibit osteoclast generation and reverse aged BMSCs (Figure 6Q).

**Figure 7:**
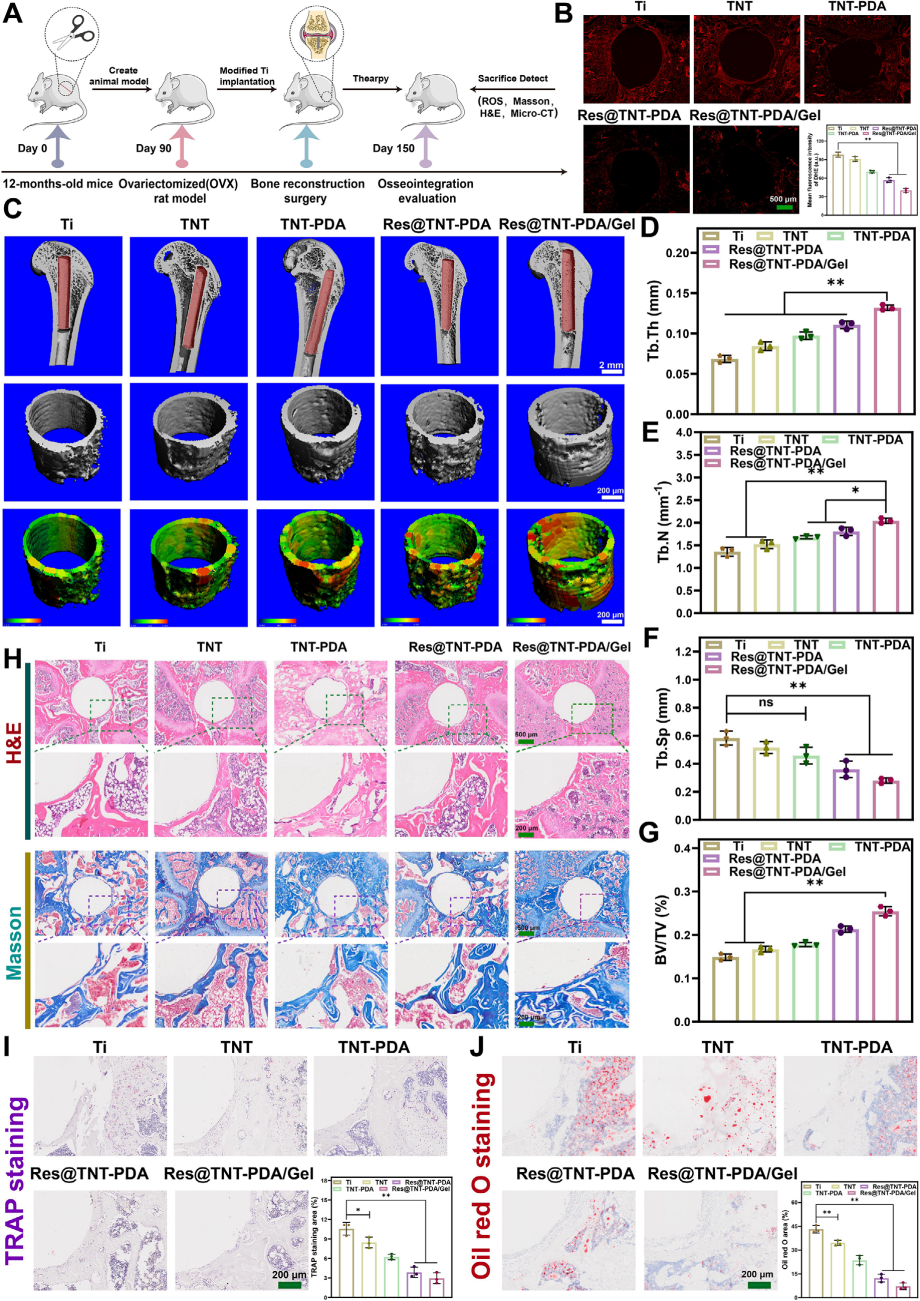
In vivo evaluation of Res@TNT‐PDA/Gel enhanced osseointegration properties. A) Schematic diagram of animal experimental therapy. B) Representative images and quantitative statistical results of bone tissue cryosections showing ROS fluorescence in various samples (n = 3). Scale bar: 500 µm. C) New bone formation and trabecular thickness assessed by micro‐CT after surgical implantation for 2 months. Scale bar: 1 mm & 200 µm. D) Quantitative analysis of trabecular thickness (Tb. Th), E) trabecular number (Tb. N), F) trabecular bone separation (Tb. Sp) and G) new bone volume per total volume (BV/TV) around various implants (n = 3). H) H&E staining and Masson staining of new bone around various Ti implants at 2 months. Scale bar: 500 and 200 µm. I) TRAP staining image and quantitative analysis of bone tissue around various Ti implants at 2 months (n = 3). Scale bar: 200 µm. J) Oil red O staining image and quantitative analysis of bone tissue around various Ti implants at 2 months (n = 3). Scale bar: 200 µm. Data were presented as mean values ± standard deviations (SD); error bars = SD. *p < 0.05, **p < 0.01.

The in vivo osseointegration effects of Ti, TNT, TNT‐PDA, Res@TNT‐PDA, and Res@TNT‐PDA/Gel were evaluated by establishing animal models of senile osteoporosis (**Figure** **7**A). To ascertain the impact of modified titanium on ROS scavenging in vivo, ROS levels in the bone tissue surrounding the implant were quantified using a DHE fluorescence probe 7 days post‐implantation. The ROS fluorescence intensity of the Ti and TNT groups was found to be higher than that of the other groups, while the immunofluorescence intensity of the Res@TNT‐PDA/Gel group was observed to be the lowest (Figure 7B). The findings demonstrated that the modified titanium exhibited an exceptional capacity for ROS scavenging. The formation of new bone around the various implants was analyzed using micro‐CT. As illustrated in Figure 7C, the Res@TNT‐PDA/Gel group exhibited a greater capacity for new bone formation than the other groups. Furthermore, the results of the quantitative analysis demonstrated that the levels of BV/TV, Tb. Th and Tb. N in the Res@TNT‐PDA/Gel group was markedly higher than those observed in the other groups (Figure 7D–G), while the levels of Tb. Sp exhibited a significant reduction (Figure 7F). The formation of new bone at the interface of the different implants was evaluated using H&E and Masson staining. As illustrated in Figure 7H, a substantial quantity of new bone tissue was observed in the vicinity of the Res@TNT‐PDA/Gel implant. However, in the other groups, no dense new bone tissue was formed between the implant and the host tissue. The number of osteoclasts and the number of adipocytes in the vicinity of the implant were quantified through TRAP staining (Figure 7I) and Oil Red O staining (Figure 7J), respectively. The results demonstrated a decreasing trend in the number of osteoclasts and adipocytes around Ti, TNT, TNT‐PDA, Res@TNT‐PDA, and Res@TNT‐PDA/Gel in sequence.

### 2.7 Biological Mechanism Analysis

**Figure 8:**
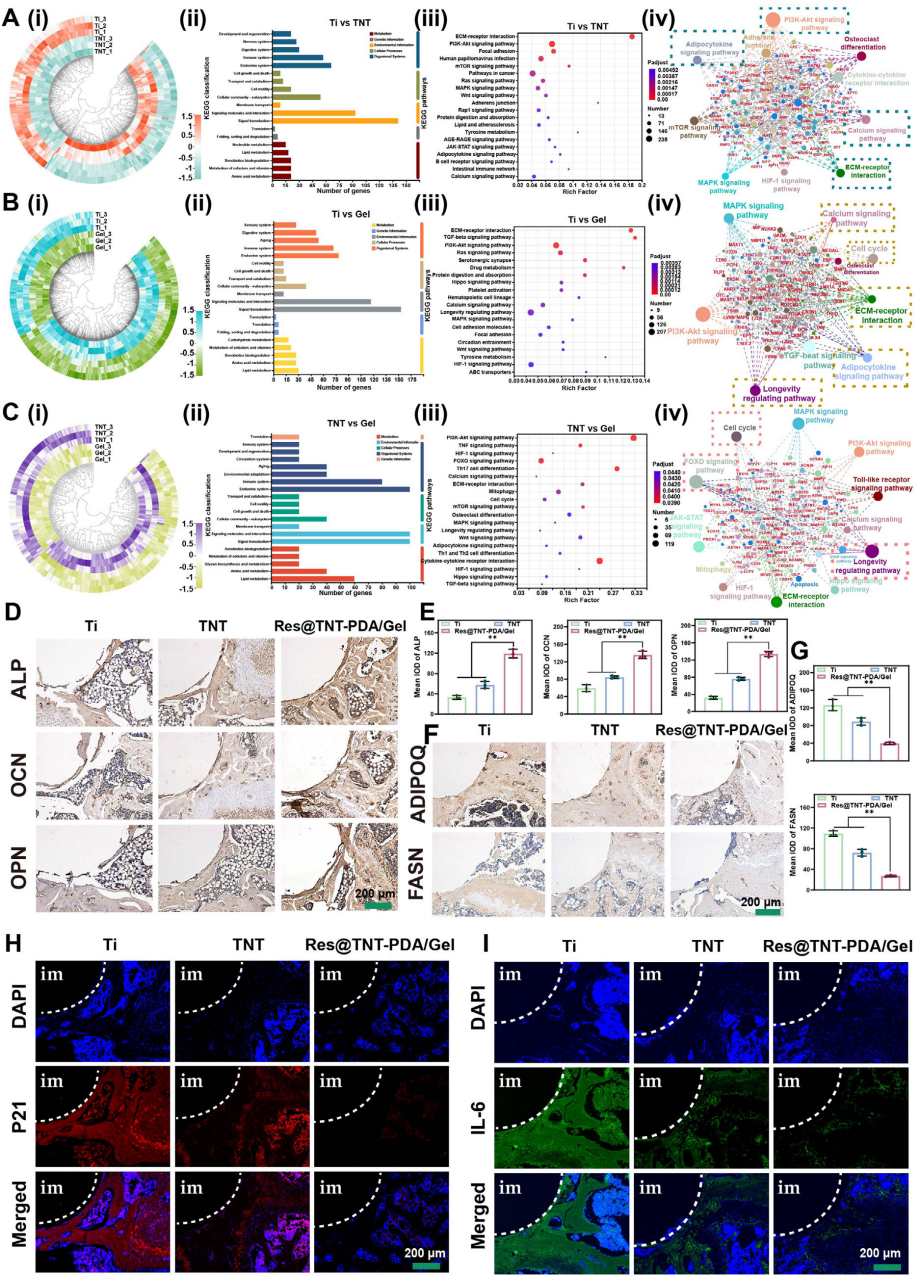
The mechanism of modified titanium was investigated using RNA‐seq results. A) Differential gene expression heatmap, KEGG pathway classification analysis, KEGG enrichment analysis, and differential expression‐associated gene pathway interaction analysis of control (Ti) and TNT groups. B) Differential gene expression heatmap, KEGG pathway classification analysis, KEGG enrichment analysis, and differential expression‐associated gene pathway interaction analysis of control (Ti) and Res@TNT‐PDA/Gel groups. C) Differential gene expression heatmap, KEGG pathway classification analysis, KEGG enrichment analysis, and differential expression‐associated gene pathway interaction analysis of TNT and Res@TNT‐PDA/Gel groups. D) Immunohistochemical staining and E) average optical density of ALP, OCN, and OPN of new bone around modified Ti implants (n = 3). F) Immunohistochemical staining and G) average optical density of ADIPOQ and FASN of new bone around modified Ti implants (n = 3). H) P21 immunofluorescence image of the representative section around the implant. I) IL‐6 immunofluorescence image of the representative section around the implant. Data were presented as mean values ± standard deviations (SD); error bars = SD. *p < 0.05, **p < 0.01.

To gain further insight into the mechanism of action of modified titanium implants on aged BMSCs, we conducted a transcriptomic analysis. The analysis yielded a total of 30560 expressed genes. The proportion of bases with a quality score of Q30 or above was more than 94.94%. In particular, the mismatch rate (Figure S10A, Supporting Information), base content (Figure S10B, Supporting Information), expression distribution (Figure S10C, Supporting Information), and saturation curve (Figure S10D, Supporting Information) demonstrate that the sequencing results are reasonable. Furthermore, the principal component analysis (PCA) and correlation analysis between samples provide additional evidence for the reliability of the data (Figure S11, Supporting Information). The results of the differential analysis demonstrated significant alterations in gene expression between the distinct sample groups (Figure S12A, Supporting Information). The volcano map results further demonstrated the specific changes in differential genes (Figure S12B, Supporting Information). A detailed GO enrichment analysis, KEGG pathway analysis, and protein interaction analysis of the differential gene expression revealed that the osteogenic differentiation signaling pathway (PI3K‐Akt signaling pathway, Calcium signaling pathway, ECM‐receptor interaction, etc.) was the most abundant in the TNT and Res@TNT‐PDA/Gel groups compared to the Ti group (**Figure** **8A,B**; Figures S13 and S14, Supporting Information). Moreover, the TNT and Res@TNT‐PDA/Gel groups demonstrated regulatory effects on the signaling pathway associated with adipogenic differentiation. Furthermore, in comparison to the TNT group, the Res@TNT‐PDA/Gel group focused mainly on the regulation of aging‐related signaling pathways, including the cell cycle, the FOXO signaling pathway, and the longevity regulating pathway (Figure 8C).

ALP, OCN, and OPN are recognised markers of osteogenesis and are among the most commonly used indicators for the evaluation of osteogenic differentiation. The protein levels of ALP, OCN, and OPN in the tissue were detected by immunohistochemical staining, and it was found that the Res@TNT‐PDA/Gel group exhibited significantly positive staining for ALP, OCN, and OPN (Figure 8D,E). Concurrently, the identification of ADIPOQ and FASN proteins linked to adipogenesis differentiation revealed that both ADIPOQ and FASN were markedly diminished in the Res@TNT‐PDA/Gel group (Figure 8F,G). Moreover, the fluorescence levels of P21 and IL‐6 were evaluated in the bone marrow microenvironment surrounding the implants, and P21 and IL‐6 expressions were found to be significantly lower in the Res@TNT‐PDA/Gel group than in the Ti and TNT groups (Figure 8H; Figure S15A, Supporting Information). The results of the SASP detection demonstrated that Res@TNT‐PDA/Gel was capable of significantly inhibiting the secretion of the SASP factor (Figure 8I; Figure S15B, Supporting Information).

**Figure 9:**
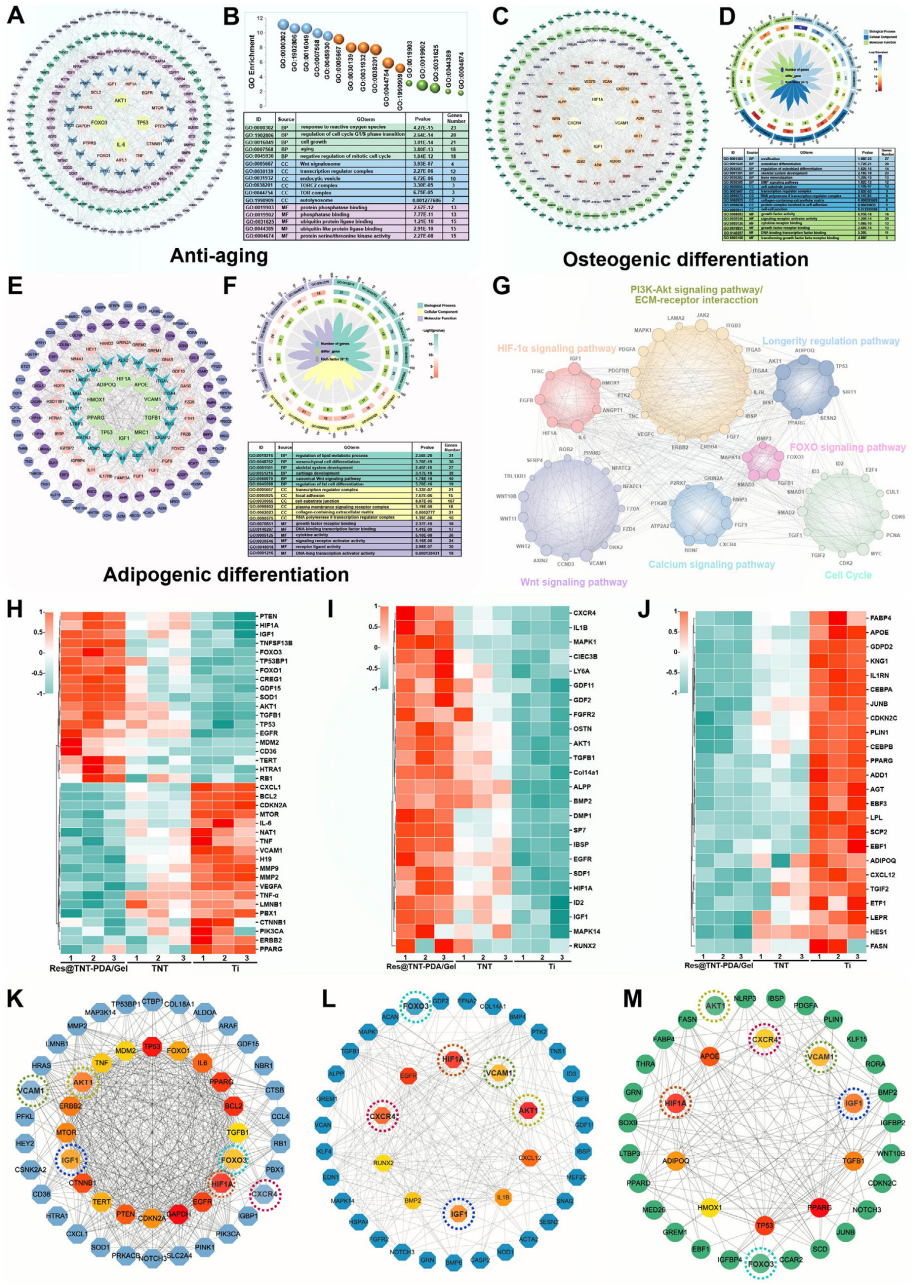
The bioinformatics analysis of the mechanism of action of modified titanium. A) Protein interaction analysis of anti‐aging related genes. B) GO enrichment analysis of genes related to anti‐aging. C) Protein interaction analysis of osteogenic differentiation‐related genes. D) GO enrichment analysis of genes related to osteogenic differentiation. E) Protein interaction analysis of adipogenic differentiation‐related genes. F) GO enrichment analysis of genes related to adipogenic differentiation. G) Analysis of the differentially expressed genes associated with the pathways and their protein interactions. Heatmaps of differential genes associated with anti‐aging H), osteogenic differentiation I), and adipogenic differentiation. J) The interaction network of core proteins involved in anti‐aging K), osteogenic differentiation L), and adipogenic differentiation (M).

A comparative analysis of the common genes associated with aging, osteogenic, and adipogenic differentiation among three differentially expressed genes was conducted to further investigate the mechanisms of action of TNT and Rev in the Res@TNT‐PDA/Gel group. The protein‐protein interaction network and Matthews correlation coefficient algorithms were employed to analyze hub genes to elucidate the significance of key genes in the regulation of aging and osteogenic/ adipogenic differentiation (**Figure** **9**A–F). Our findings revealed that a number of the same genes act in concert to regulate pivotal signaling pathways (Figure 9G; Figure S14, Supporting Information) associated with the processes of aging, osteogenesis, and adipogenic differentiation, such as the PI3K‐Akt signaling pathway, the longevity regulation pathway, the HIF‐1 signaling pathway, the FOXO signaling pathway, cell cycle, calcium signaling pathway, and the Wnt signaling pathway. These key signaling pathways are intimately associated with oxidative stress, the cell cycle, cell proliferation, and cell differentiation of BMSCs in the context of the aging microenvironment. By extracting the expression levels of core genes with strong correlations to aging, osteogenesis, and adipogenic differentiation, we can observe the expression of these genes in three distinct materials using heat maps (Figure 9H–J). Some longevity genes, liking FOXO1, FOXO3, SOD, are highly expressed in the Res@TNT‐PDA/Gel group, while some inflammatory genes (IL‐1, IL‐6, TNF‐α, MMP9, etc.) are highly expressed in the Ti group. In comparison with the other two groups, the Res@TNT‐PDA/Gel group exhibited a high level of osteogenic gene expressions (BMP2, BMP4, ALPP, COL, RUNX2, etc.) and a low level of adipogenic gene expressions (APOE, PPARG, ADIPOQ, FASN, etc). The interaction network of core proteins involved in aging (Figure 9K), osteogenic differentiation (Figure 9L), and adipogenic differentiation (Figure 9M) demonstrated that certain key proteins, including FOXO3, HIF‐1α, IGF1, CXCR4, VCAM1, and AKT, exert a significant influence on both the aging, adipogenesis, and osteogenic differentiation of BMSCs.

**Figure 10:**
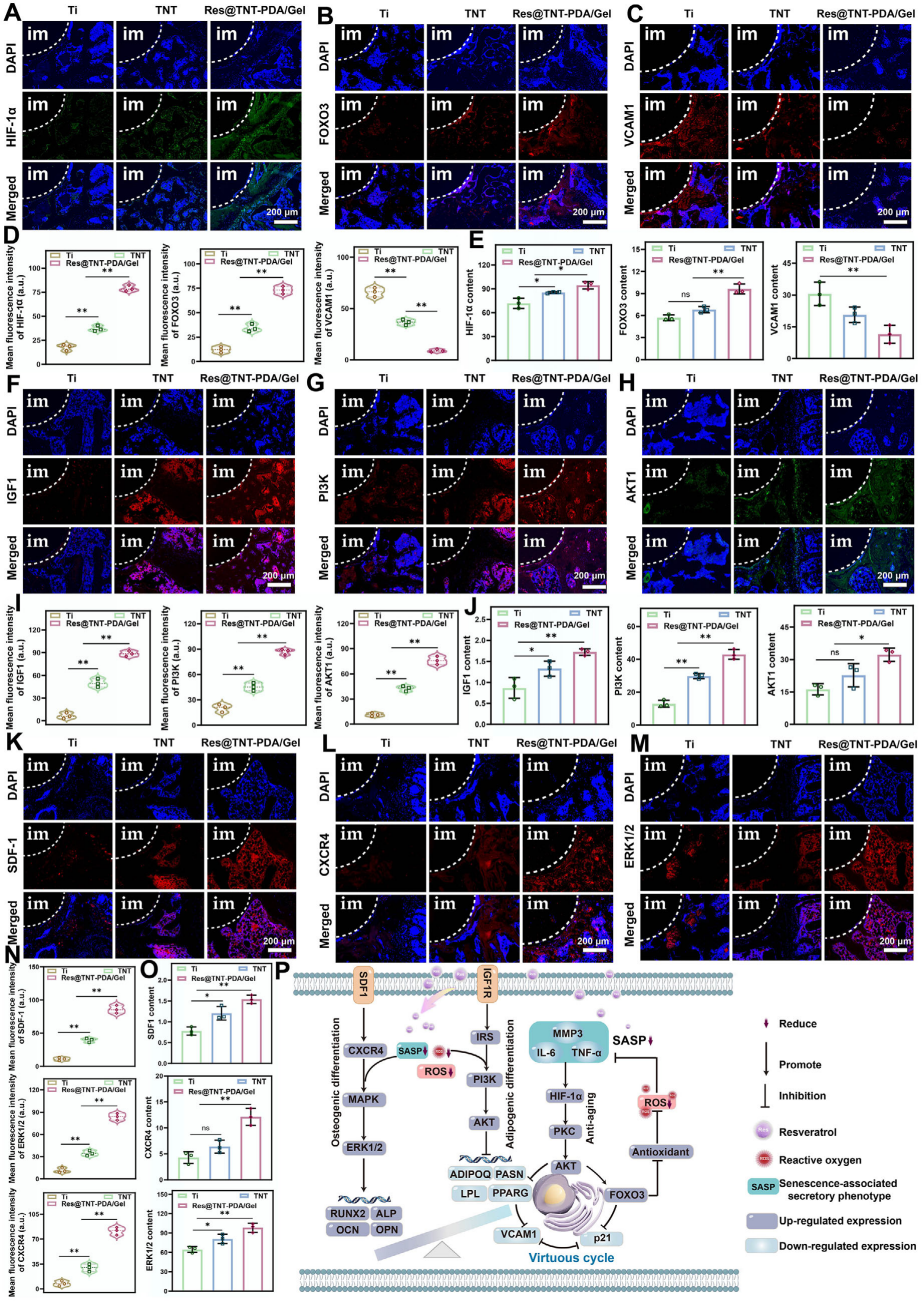
The molecular mechanism of Res@TNT‐PDA/Gel in senile osteoporosis. A)HIF‐1α immunofluorescence image of the representative section around the implant. B) FOXO3 immunofluorescence image of the representative section around the implant. C) VCAM1 immunofluorescence image of the representative section around the implant. D) Quantification of relative fluorescence intensity of HIF‐1α, FOXO3, and VCAM1. E) The expression of HIF‐1α, FOXO3, and VCAM1. F) IGF1 immunofluorescence image of the representative section around the implant. G) PI3K immunofluorescence image of the representative section around the implant. H) AKT1 immunofluorescence image of the representative section around the implant. I) Quantification of relative fluorescence intensity of IGF1, PI3K, and AKT1. J) The expression of IGF1, PI3K, and AKT1. K) SDF‐1 immunofluorescence image of the representative section around the implant. L) CXCR4 immunofluorescence image of the representative section around the implant. M) ERK1/2 immunofluorescence image of the representative section around the implant. N) Quantification of relative fluorescence intensity of SDF‐1, CXCR4, and ERK1/2. O) The expression of SDF‐1, CXCR4, and ERK1/2. P) Schematic diagram of the molecular mechanism of Res@TNT‐PDA/Gel in senile osteoporosis. Data were presented as mean values ± standard deviations (SD); error bars = SD. *p < 0.05, **p < 0.01.

The expression of these key proteins was analyzed in different groups at the RNA, protein, and tissue levels (**Figure** **10**A–O). Among the nine core proteins, the expression of eight proteins (FOXO3, HIF‐1α, IGF1, CXCR4, PI3K, AKT, SDF‐1, and ERK1/2) increased sequentially from the Ti, TNT, and Res@TNT‐PDA/gel groups. Conversely, vascular cell adhesion molecule 1 (VCAM1) demonstrated an inverse trend.

## 3 Discussion

Senile osteoporosis is a metabolic bone disease that is associated with the aging process. As age increases, there is a concomitant decrease in bone density, structure, and function.^[^ ^1^, ^8^, ^21^ ^]^ Furthermore, an elevated risk of fracture is accompanied by a prolonged healing process in senile osteoporosis.^[^ ^22^ ^]^ It is widely accepted that the diminished immune system characteristic of the elderly is the primary factor influencing the process of fracture healing. The skeleton is a dynamic and uninterrupted tissue, and it is composed of various tissues including bone, cartilage, fat, etc. which are precisely regulated by corresponding stem cells, such as hematopoietic stem cells (HSCs) and mesenchymal stem cells (MSCs) that determine the homeostasis of bone. Hematopoietic lineage cells and mesenchymal lineage cells are highly heterogeneous cell populations composed of distinct subpopulations. Hematopoietic lineage cells, including macrophages, neutrophils, and natural killer cells, play a crucial role in maintaining immune stability.^[^ ^1^, ^7^, ^11^, ^12^, ^23^ ^]^ Mesenchymal lineage cells are capable of undergoing tri‐lineage differentiation, which encompasses the formation of chondrocytes, osteoblasts, and adipocytes.^[^ ^2^, ^14^ ^]^ This process is essential for maintaining bone homeostasis and facilitating fracture healing.

HSCs and MSCs have the potential for self‐renewal and multi‐directional differentiation, which can maintain cell renewal and tissue repair when mature cells in the body die due to aging. But stem cells themselves can also age.^[^ ^24^ ^]^ The aging of stem cells can affect the overall aging of the body. All aging phenomena – tissue degeneration – can be explained as signs of aging at the level of stem cells in the body.^[^ ^2^, ^10^, ^25^ ^]^ Although in vitro cell culture is commonly used to study stem cells, target cells may not always exhibit similar behavior in natural biological environments due to significant differences between culture conditions and *in v*ivo conditions.^[^ ^19^ ^]^

The present study employs single‐cell transcriptomic analysis to elucidate the dynamics of skeletal stem cells in Sprague‐Dawley rats across a range of ages. This approach allows for the identification and characterization of rare cell groups, thereby facilitating the inference of their self‐renewal and differentiation processes. It is a well‐established fact that aging is a common phenomenon in nature.^[^ ^2^, ^11^ ^]^ Consequently, HSCs and MSCs in the bone marrow also undergo the aging process. However, our findings indicate that the aging dynamics and characteristics of HSCs and MSCs exhibit distinct patterns with advancing age. In the third month, hematopoietic lineage cells first undergo age‐related changes, resulting in a notable decline in cell number. The aged HSCs lead to increased production of myeloid cells (e.g., megakaryocytes and neutrophils) at the expense of lymphoid cells (e.g., T cells and B cells), accompanied by the release of interleukin‐6 (IL‐6) and tumor necrosis factor‐α (TNF‐α) (Figure 1I). At 16 months, the differentiation of myeloid cells from hematopoietic stem cells becomes more pronounced. Except for megakaryocytes and neutrophils, the majority of cells exhibited a reduction in cell number. This myeloid bias results in a weakened immune response. Furthermore, IL‐6 and TNF‐α were released by aged neutrophils and megakaryocytes, respectively. Decrease in the number of hematopoietic lineage cells at 3 and 16 months due to HSC aging. The most irreversible cause of HSC aging is related to the accumulation of random DNA damage. The accumulation of DNA damage, decreased cyclin‐dependent kinases, cell cycle arrest, and reduced cell proliferation are hallmarks of the aging HSCs.^[^ ^26^ ^]^ Additionally, these aging cells secrete increased levels of SASP mediators and proteases, which promote inflammation and have elevated intracellular reactive oxygen species (ROS) levels. With age, the differentiation potential of HSC diminishes with each cell division, ultimately leading to the depletion of hematopoietic stem cells.^[^ ^27^ ^]^ In the case of the mesenchymal lineage cells (Figure 2A), at 16 months, there was a notable increase in the number of adipocytes and a corresponding decrease in the number of chondrocytes. Moreover, the number of LCP, OB, and Ocy has remained relatively constant, whereas a slight decrease was observed in the number of EMP, IMP, and LMP. The reduction in the number of HSCs and MSCs is attributable to the degradation of cyclin‐dependent kinases, cell cycle arrest, and a decline in cell proliferation, which are all consequences of cellular aging. These findings suggest that as age increases, there is a concomitant decrease in bone immune function and osteogenic ability.

Second, aged HSCs and MSCs exhibit indications of cellular senescence, characterized by a reduction in cyclin‐dependent kinases (e.g., CDK2, CDK4, and CDK6) with age, and an irreversible growth arrest. This is exemplified by the inhibition of cell cycles in the G0/G1 phase. However, the SASP (comprising IL‐6, TNF‐α, and MMP9) secreted by the various age groups of hematopoietic and mesenchymal lineage cells differs. The secretion of the SASP is more prevalent among cells of the hematopoietic lineage than among those of the mesenchymal lineage. Moreover, the SASP is predominantly secreted by neutrophils and MK cells within the hematopoietic lineage, whereas it is primarily secreted by adipocytes within the mesenchymal lineage. As illustrated in Figure 2M, with the progression of time, hematopoietic lineage cells initially undergo a process of aging, which is primarily evidenced by the exhaustion of HSCs. On the one hand, the quiescent period of hematopoietic lineage cells is disrupted, and the excessive myeloid differentiation of HSCs can result in their exhaustion. On the other hand, aged HSCs generate excess reactive oxygen species (ROS) within the mitochondrial compartment and secrete a substantial quantity of SASP factors, resulting in the aging of MSCs.

The aging of MSCs leads to a decline in their regenerative and differentiation potential.^[^ ^2^, ^28^ ^]^ Aged MSCs demonstrate a differentiation bias, frequently favoring adipogenesis (fat cell formation) over osteogenesis (bone cell formation) and chondrocytes (cartilage cells). This shift contributes to age‐related bone loss and osteoarthritis. This is because a greater number of transcription factors regulate adipogenic differentiation than osteoblastic differentiation (Figure 2F). The SASP factors and ROS secreted by aging HSCs could promote aging and adipogenic differentiation of MSCs. Meanwhile, the differentiated adipocytes secreted a substantial quantity of SASP factors, which further intensified the trend of MSCs aging and adipogenic differentiation, thereby precipitating a vicious cycle wherein the aging state of MSCs (Figure 2M). Bone marrow was extracted from SD rats of different ages according to the single‐cell data, whose immunohistochemistry and immunofluorescence results verified the results of single‐cell analysis. As age increases, cells within the bone marrow undergo the aging process, resulting in the production of ROS and SASP factors (Figure 3A–I). These factors can contribute to an increase in adipocytes and osteoclasts, a decrease in osteoblasts (Figure 3B–E), and ultimately lead to the development of senile osteoporosis (Figure 3K). Unlike conventional forms of osteoporosis, the expression of the aging marker p21 is significantly elevated in individuals with senile osteoporosis (Figure 3G).

The potential pathological mechanism of senile osteoporosis was further elucidated through the analysis of clinical data. The differential expression of miRNAs in plasma samples from senile patients with and without osteoporosis was initially examined. A total of 220 differentially expressed miRNAs (Figure 3N) and their 512 hub target genes (Figure 3O,P) were identified. The core 50 genes (including STAT3, VEGFA, HIF‐1A, TP53, JUN, MYC, CTNNB1, IL‐6, SRC, CASP3, etc.) are closely associated with the processes of aging, oxidative stress, and bone metabolism (Figure 3Q–T). The most significantly affected target genes were found to regulate several key pathways, including the PI3K‐Akt signaling pathway, osteoclast differentiation, the mTOR signaling pathway, the longevity regulation pathway, the HIF‐1α signaling pathway, the FOXO signaling pathway, autophagy, the TNF signaling pathway, and others. These pathways are intimately associated with aging, osteoporosis, and bone homeostasis. Consequently, based on these 50 key genes, resveratrol (Res) was selected as the most correlated with the core target genes (Figure 3U–W), which is a non‐flavonoid polyphenolic compound derived from plants.

Ti alloys are widely used in hard tissue repair. However, when used in a patient with senile osteoporosis, the aging skeletal microenvironment can result in poor bonds between bone tissue and Ti implants.^[^ ^17^, ^29^ ^]^ In our previous research, we discovered that Ti with micro‐nano surface morphology could enhance the osteointegration effect, but it also had the unintended consequence of accelerating the aging of BMSCs.^[^ ^17^ ^]^ To reverse the aging process and promote osteogenic differentiation of MSCs, resveratrol was loaded into the nanotubes on the Ti surface and sealed with a two‐group hydrogel network coating comprising methacrylate‐modified gelatin and carboxylated chitosan. This may result in a reduction in the release rate of resveratrol and an associated decrease in its toxicity due to a delayed release.

Resveratrol is a polyphenolic compound with antioxidant, anti‐inflammatory, and potential anti‐aging properties.^[^ ^30^ ^]^ The in vitro results demonstrated that, in the high ROS environment associated with osteoporosis, the prepared titanium implant enables the gradual release of resveratrol (Figure 4K), which in turn reduces oxidative damage (ROS, Figure 5E) and lysosomal changes (SA‐β‐gal, Figure 5F) caused by MSCs aging. Concurrently, the G2/M phase of MSCs is augmented (Figure 5D), thereby enhancing their vitality and proliferation (Figure 5A). This suggests that the aging state of MSCs has been changed. The alteration in the state of aged stem cells has led to a reversal of the differentiation trends associated with osteogenesis and adipogenesis. All four groups of nanotubes (TNT, TNT‐PDA, Res@TNT‐PDA, and Res@TNT‐PDA/Gel) exhibited enhanced osteogenic differentiation capabilities, particularly the Res@TNT‐PDA/Gel group, in comparison to the pure titanium group (Figure 6). Conversely, genes associated with adipogenic differentiation (PPARγ, FASN, FABP4, CEBPA, and LPL) (Figure 6K) and factors linked to osteoclasts (CTSK, OSM, NFATC1, RANK, and ACP5) (Figure 6P) exhibited a notable decline in the last four groups. Based on the results of aging and differentiation, we conclude that Ti with nanotubes may not reduce MSCs aging compared to pure Ti, but it may promote osteogenic differentiation of MSCs through contact guidance. However, after the introduction of resveratrol, it significantly reduces oxidative stress (Figure 5E) and SASP factors (Figure 5J) induced by the aging process, thereby facilitating a switch from adipogenic differentiation of stem cells to osteogenic differentiation. At the same time, it could reduce osteoclastogenesis, which in turn increases bone density and bone integration. Thus, the ability of Res@TNT‐PDA/Gel to facilitate bone differentiation is enhanced, while the potential for adipogenic differentiation and osteoclastogenesis is reduced. The Micro‐CT, H&E, Masson's stain, and immunofluorescence results (Figure 7) from animal experiments supported our hypothesis. The results showed that the modified titanium exhibited exceptional ROS scavenging capacity and that the Res@TNT‐PDA/Gel group could significantly enhance the effect of bone integration around the implant in the senile osteoporosis model with bone defect.

The three most representative groups (Ti, TNT, and Res@TNT‐PDA/Gel) were selected for further analysis. The results of the RNA sequencing demonstrated that, in comparison to the pure Ti group, the differentially expressed genes (Figure 8A) in the TNT group predominantly influence the osteoblasts‐osteoclasts equilibrium pathways, including the PI3K‐Akt signaling pathway, osteoclast differentiation, adipocytokine signaling pathway, calcium signaling pathway, and so forth. Moreover, the differentially expressed genes in the Res@TNT‐PDA/Gel group are primarily associated with aging pathways (Figure 8B), including longevity‐regulating pathways, FOXO signaling pathways, cell cycles, and the PI3K‐Akt signaling pathway, among others. The immunohistochemistry and immunofluorescence results demonstrated that the TNT group exhibited higher expression of osteogenic genes (ALP, OCN, and OPN) and lower expression of adipose genes (ADIPOQ and FASN) relative to the Ti group (Figure 8D–G). These trends are of particular significance within the Res@TNT‐PDA/Gel group. Conversely, the Ti group displayed elevated levels of SASP factors (P21 and IL‐6), which exhibited a notable decline in the TNT group and were rarely detected in the Res@TNT‐PDA/Gel group. So, the number of osteoclasts and adipocytes around Ti, TNT, TNT‐PDA, Res@TNT‐PDA, and Res@TNT‐PDA/Gel showed a decreasing trend in sequence (Figure 7I,J).

Among the nine core proteins (Figure 10), VCAM1 plays a pivotal role in the pathogenesis of inflammation and immune responses, functioning as a mediator of leukocyte adhesion to the vascular endothelium.^[^ ^31^ ^]^ The expression of VCAM1 tends to increase with age, which represents a crucial factor in the intersection of aging and inflammation. These include HIF‐1α, FOXO3, and VCAM1, which are principal proteins in the longevity regulation signaling pathway. They regulate cell adhesion, the cell cycle, and proliferation. The main proteins in the PI3K/AKT signaling pathway are IGF1 and AKT. The main proteins in the PI3K/AKT signaling pathway are IGF1 and AKT. PI3K is a cytoplasmic lipid kinase that is activated by upstream growth factors. The activated PI3K product, phosphatidylinositol triphosphate, can bind with the serine/threonine kinase AKT, which results in AKT translocating from the cytoplasm to the cell membrane. Concurrently, the conformation alterations result in the phosphorylation of AKT serine and threonine residues.^[^ ^32^ ^]^ Activated AKT can subsequently activate downstream target proteins and participate in the growth, differentiation, and other processes of cells, including MSCs. SDF‐1 can also activate the homologous CXCR4 receptor to promote the migration, proliferation, and osteogenic differentiation of BMSCs.

The findings of Figures 5, 6, 8, 9, 10 suggest that the construction of titanium nanotubes on the surface of titanium may potentially mitigate the effects of SASP factors in the context of age‐related osteoporosis, retard the aging process of BMSCs, facilitate the differentiation of BMSCs into osteoblasts, and inhibit the differentiation of BMSCs into adipocytes. The introduction of resveratrol to the implant significantly reverses the aging state of BMSCs, thereby enhancing their osteogenic differentiation performance and inhibiting their adipogenic differentiation performance. The potential mechanism of action is illustrated in Figure 10P. Modified titanium implants have been demonstrated to facilitate bone integration in the context of age‐related osteoporosis through three principal mechanisms. On the one hand, the released resveratrol can reduce reactive oxygen species (ROS) in the aging microenvironment and bind to insulin‐like growth factor 1 (IGF1) receptors on the cell membrane surface, promoting the expression of the phosphatidylinositol 3‐kinase (PI3K)/protein kinase B (AKT) signaling pathway. This, in turn, inhibits the expression of proteins involved in the differentiation of adipose tissue, including adiponectin (ADIPOQ), fatty acid synthase (FASN), peroxisome proliferator‐activated receptor gamma (PPARγ), and lipoprotein lipase (LPL), thereby inhibiting the differentiation of bone marrow‐derived mesenchymal stem cells (BMSCs) into adipose tissue. Second, the released resveratrol can also reduce the SASP factor in the aging microenvironment, thereby restoring the blocked cell cycle. This is achieved by promoting the expression of HIF‐1a and FOXO3 while inhibiting the expression of VCAM1 and the aging factor P21. Thirdly, the nanotube structure on the surface of the implant and resveratrol can activate the SDF‐1/CXCR4 signaling axis in BMSCs, thereby promoting the expression of osteogenic‐related proteins (RUNX2, ALP, OCN, OPN) in BMSCs through the MAPK/ERK signaling pathway. By reversing the aging microenvironment and promoting the osteogenic differentiation of BMSCs, the generation of adipocytes is inhibited, and the skeletal microenvironment of the aging osteoporosis in Figure 2M is transformed from ‘a vicious cycle’ to ‘a virtuous cycle’, thereby improving bone homeostasis and promoting bone integration. This study employs single‐cell transcriptome sequencing (scRNA‐seq) technology to facilitate the labeling of rare cell populations within bone stem cells, thereby enabling the inference of their differentiation processes. The combination of transcriptome sequencing methods with other techniques offers a more accurate foundation for the treatment of senile osteoporosis and the development of related bone repair materials.

## 4 Conclusion

In this study, we utilized a combination of rat single‐cell transcriptome analysis and human serum transcriptome analysis to explore the cellular subpopulations and dynamics of bone stem cells in age‐related osteoporosis. Our findings guided the design of titanium implants tailored for individuals with aging osteoporosis. The results revealed distinct aging characteristics in HSCs and MSCs. Aged HSCs produce higher levels of ROS and secrete more SASP factors that contribute to MSC aging. Aged MSCs tend to differentiate into adipocytes more than osteoblast, creating a cycle that further accelerates MSC aging. We screened out key genes associated with aging osteoporosis and selected resveratrol as a potential drug. Resveratrol was incorporated into titanium implants, and sealed with a ROS‐responsive hydrogel coating to realize a gradual release within the body. This innovative approach reduced oxidative damage and secretion of SASP factors caused by aged MSCs and promoted a shift in stem cell differentiation from adipocyte to osteoblast. At the same time, osteoclastogenesis was reduced. So, the skeletal microenvironment of the aging osteoporosis is transformed from a negative feedback loop to a positive feedback loop, thereby improving bone homeostasis and promoting osseointegration of the Ti implant. The combination of transcriptome sequencing methods used in this work offers a more accurate foundation and inspiration for the treatment of senile osteoporosis and the development of related bone repair materials.

## Conflict of Interest

The authors declare no conflict of interest.

## Author Contributions

W.F., W.Y., and K.C. performed conceptualization, wrote the original draft, and wrote, reviewed, and edited the draft. W.F., M.M., P.G., Y.Y., R.W., and Y.Y. performed methodology. W.F., T.Z., M.M., Y.Z., and T.Y. performed investigation. W.F. and T.Z. performed visualization. R.B., W.Y., X.Y., and K.C. performed supervision.

## Supporting information



Supporting Information

## Data Availability

The data that support the findings of this study are available from the corresponding author upon reasonable request.
