# Supplementary material for: A Study of Skeletal Stem Cell Dynamics and Its Potential Applications in the Design of a Titanium Implant for Senile Osteoporosis
Source: Adv Sci (Weinh). 2025 Jun 19;12(34):e06982. doi: 10.1002/advs.202506982 (PMC12442682; doi:10.1002/advs.202506982)
Supplement: Supplementary file 1 — Supporting Information [file ADVS-12-e06982-s001.docx]

Supplementary Materials for

**A study of skeletal stem cell dynamics and its potential applications in the design of a titanium implant for senile osteoporosis**

Wuzhe Fan,^1^ Tao Zheng,^1^ Mingsong Mao,^3^ Pengfei Gao,^1^ Yulu Yang,^1^ Rong Wang,^1^ Yao Yang,^1^ Yangpeng Zuo,^1^ Tiantian Yuan,^1^ Ruqing Bai,^2^*Weihu Yang,^1^* Xingchen Yan,^4^* Kaiyong Cai,^1^*

**Affiliations**

^1^ Key Laboratory of Biorheological Science and Technology, Ministry of Education College of Bioengineering, Chongqing University, Chongqing, 400044, China.

^2^ State Key Laboratory of Mechanical Transmission for Advanced Equipment, Chongqing University, Chongqing, 400044, China.

^3^ School of Basic Medical Sciences, Anhui Medical University, Hefei, 230032, China.

^4^ Institute of New Materials, Guangdong Academy of Sciences, Guangdong-Hong Kong Joint Laboratory of Modern Surface Engineering Technology, Guangdong Provincial Key Laboratory of Modern Surface Engineering Technology, Guangzhou, Guangdong 510651, China

*corresponding author. email: Ruqing Bai ([ruqing.bai@cqu.edu.cn](mailto:ruqing.bai@cqu.edu.cn)), Weihu Yang ([yangweihu@cqu.edu.cn](mailto:yangweihu@cqu.edu.cn)), Xingchen Yan (yanxingchen@gdinm.com) and Kaiyong Cai ([kaiyong_cai@cqu.edu.cn](mailto:kaiyong_cai@cqu.edu.cn))

**This PDF file includes:**

Supplementary Text

Figs. S1 to S15

Tables S1 to S4

Supplementary Text

1. **Materials and Methods**

**Materials**

Commercial Titanium slices and titanium nails (diameter is 1.5 mm) were provided from Northwest Institute for Non-ferrous Metal Research Institute (Shanxi, China). Resveratrol (Res), 4-(2- Aminoethyl) benzene-1,2-diol, N-hydroxysuccinimide (NHS), N-(3-dimethylaminopropyl)-N′-ethylcarbodiimide hydrochloride (EDC), Methacrylic anhydride were purchased from Macklin Co. Ltd (Shanghai, China). 2,2'- [propane-2,2-diylbis (thio)] diacetic acid was obtained from Bidepharm Co. Ltd (China). Carboxylated chitosan, Gelatin were purchased from Aladdin Co. Ltd (China). Bicinchoninic acid (BCA) and alkaline phosphatase (ALP) were received from Nanjing Jiancheng Biotechnology Institute (Nanjing, China). The RNA extraction and Prime Script RT reagent kits were obtained from Takara (Dalian, China). Primer synthesis services were provided by Sangon Biotech (Shanghai, China). The primary antibodies were provided from Abcam Co. Ltd (UK). All other chemical reagents were purchased from Chuandong Chemical Reagent Co. Ltd (analytical reagent grade).

**Design and characterization of Titanium substrates**

**Surface modification of titanium**

Titanium slices (10 mm × 10 mm × 1 mm) and nails (diameter: 1.5 mm; length: 10 mm) were used for in vitro and in vivo experiments severally. Titanium dioxide nanotubes were synthesised in accordance with previous studies. Briefly, after sequential machining with sandpapers (400-2000 mesh) and ultrasonic cleaning with ethanol and distilled water, pure Titanium was treated with a voltage of 30 V in an electrolyte containing ammonium fluoride, distilled water and glycerine, and the resultant samples were named as TNT. After ultrasonic cleaning, TNT was placed in a dopamine solution (2 mg/mL) containing Tris-HCl buffer (10 mM, pH 8.5) and allowed to oscillate overnight at room temperature in the absence of light. The obtained samples were thoroughly rinsed with distilled water. The prepared Res solution (400 μg/mL) was dropped onto each substrate and vacuumed, and the samples were named Res@TNT-PDA.

**Fabrication of ROS-responsive coating on surface modification of titanium**

Synthesis of GelMA: Gelatin (10 g) was added to 100 mL of distilled water at 55 °C for 30 minutes. Then 1 mL of methacrylic anhydride was added dropwise. After reacting for 3 h, the resulting reaction mixtures were dialysed for 5 days and dried under vacuum, and the samples were named GelMA.

After reaction for 3 h at room temperature, the carboxyl groups of 2,2'-[propane-2,2-diylbis(thio)] diacetic acid (0.15 mM) were activated in N-(3-dimethylaminopropyl)-N′-ethylcarbodiimide hydrochloride (0.3 mM) and N-hydroxysuccinimide (0.3 mM) DMSO solutions. GelMA (20% w/v) and carboxylated chitosan (10 mg/ml) were then added to the activated 2,2'-[propane-2,2-diylbis(thio)] diacetic acid (0.08% v/v), and the resulting mixtures were completely covered on the surface of Res@TNT-PDA and incubated at 37℃ for 24 h to obtain Res@TNT-PDA/Gel.

**Characterization of ROS-responsive coating on surface modification of titanium**

Nuclear magnetic resonance (NMR, DD2 600 MHz, Agilent, USA) and Fourier transform infrared spectroscopy (FTIR, Thermo Fisher Scientific, USA) were used to confirm the successful synthesis of GelMA. The morphological changes of the different sample preparations were observed by environmental scanning electron microscopy (SEM, Thermo Scientific, USA). Atomic force microscopy (AFM, Dimension, Bruker, Germany) and water contact angle analysis (Model 200, Future Scientific Co., Tai Wan, China) were used to detect the roughness and surface hydrophilicity of the ROS responsive coating. The adhesion of the hydrogel coating to various substrates was tested using a universal testing machine. Briefly, the overlapping areas (1 cm × 1 cm) between titanium or modified titanium (1 cm × 5 cm) were evenly coated with a ROS-responsive hydrogel coating for the lap shear test. At the same time, porcine skin was used as a substrate to test the bond strength between the modified titanium and the tissue. After 24 hours of interaction between the parts of the lap at 37 ◦C, the adhesion of the coating was tested using a universal testing machine at the tensile rate of 0.1 mm/s.

**Stability and degradation of ROS-responsive coating on titanium surface modification**

Swelling: First, the ROS-responsive hydrogel coating of the modified titanium surface was weighed (M_0_), then placed in a 24-well plate and soaked with 1ml PBS on a shaking table at 37℃. The sample was removed at a specific time and the surrounding water was carefully removed with filter paper. The change in quality of the sample was the result of weight (M_t_) and calculation. The calculation of the swelling rate will be as follow：

Swelling rate (%) = M_t_/M_0_ × 100%

Degradation: The degradation test begins when the ROS-responsive hydrogel layer reaches maximum swelling mass. In a shaker at 37℃, 1 ml of PBS with or without H_2_O_2_ (500 nM) was added. After a set incubation time, the sample was weighed. The weight loss (Wdr) of the coating was the representation of the degradation rate and was calculated as follows:

W_dr_ = W_t_-W_0_, where W_0_ was the initial sample weight and W_t_ was the sample weight after various incubation times.

**Release of Res**

In order to explore the release kinetics of Res in modified titanium coatings, Res@TNT-PDA and Res@TNT-PDA/gel were immersed in 1 mL of PBS at 37 °C. After incubation for a certain time, the absorbance of the solution was measured using an ultraviolet spectrophotometer. Meanwhile, Res@TNT-PDA/gel was immersed in 1 mL PBS with or without H2O2 (500 nM) at 37℃ to investigate the ROS responsive release characteristics of Res@TNT-PDA/gel. The absorbance of the sample was measured after a specific incubation time.

**Assay for anti-oxidation**

DPPH radicals were used to test the antioxidant activity of modified titanium implants. First, the DPPH solution (0.1 mM) was prepared with 95% ethanol, and then the samples of different groups were incubated with the configured DPPH solution (1 mL), and the supernatant was collected by centrifugation. The absorbance value at 516 nm was monitored using an ultraviolet-visible spectrophotometer, and the intensity of its scavenging activity was assessed on the basis of the decrease in absorbance.

The hydrogen peroxide detection kit was used to determine the ability of different groups of samples to remove hydrogen peroxide. Briefly, the prepared 5 groups of materials were first placed in a 24-well plate, and then 200 μL of 300 mM hydrogen peroxide solution was added, which was left at room temperature for 24 hours, and then the removal ability was tested according to the Hydrogen Peroxide Detection Kit.

The total antioxidant capacity test kit (ABTS rapid method) was used to determine the total antioxidant capacity of different groups of samples.

**Analysis of scRNA-seq**

The scRNA-seq data for GSE145477 were generated from the Gene Expression Omnibus (GEO) database and included bone marrow mesenchymal lineage samples of different ages. In briefly, poor quality cells are excluded by setting parameters. The PercentageFeatureSet operation function was used to count the percentage of mitochondria and rRNA and to normalise the sample data by log normalisation. The FindVariableFeatures function was used to find highly variable genes, remove the batch processing effect between samples and aggregate the data for dimensionality reduction cluster processing. At the same time, the RunTSNE function was used to perform a T-distributed stochastic neighbour embedding (tSNE) downscaling analysis of the cells. Finally, the cells were annotated using published literature, cell marking websites and the SingleR package. The subsequent analysis was accomplished using the R software package.

**Disease data analysis**

The miRNA expression profiles of osteoporosis patients were taken from the GEO database (GSE93883). After differential analysis using R software package, target genes were found using miRBase database. On the basis of the Gene card database, the relationship between ageing and the target genes of osteoporosis was explained and the core genes were searched for. The HERB database was used for target drug screening.

**Experiment done *in vitro***

**Cell culture**

Bone marrow mesenchymal stem cells (MSCs) were obtained from ageing Sprague-Dawley osteoporotic rats (16 months old). The cells were cultured in DMEM containing low glucose and supplemented with 10% fetal bovine serum and 1% penicillin/streptomycin. The culture was maintained in a humidified atmosphere of 5% CO_2_ at 37 °C. The medium was replaced every 2 days, and MSCs from passages 2 to 4 were used in the subsequent experiments.

**Cell viability and proliferation**

The activity and proliferation of MSCs were determined using the CCK-8 assay and FDA/PI staining. First, MSCs were inoculated into 24-well plates at 2×10^4^ cells/mL and co-cultured with modified titanium for 4 and 7 days. The pre-prepared CCK-8 solution was then added and incubated for 30 minutes in the dark. Finally, supernatant absorbance was measured at OD450 nm utilising a microplate reader (Bio Rad 680, USA). The evaluation of FDA/PI staining was conducted via CLSM imaging.

**Detection of ROS intracellular and Medium**

Intracellular ROS were detected by means of a reactive oxygen species assay kit. After 5 days of cultivation, MSCs were incubated with 2′,7′-Dichlorofluorescin diacetate (DCFH-DA, 0.1%) for 20 minutes at 37℃, and subsequently washed three times with serum-free. Visualisation was performed by CLSM, and the average fluorescence intensity was calculated using Image J. Meanwhile, the hydrogen peroxide assay kit was used for the detection of ROS in the medium.

**Cell Cycle Analysis**

Briefly, to assess the cell cycle of MSCS, various samples were cultured in 6-well plates for 5 days. The trypsin-digested cells were resuspended in PBS, fixed with 70% (v/v) ethanol overnight at 4℃, and then probed with a propyl iodide and RNase A mixed staining solution, avoiding light at 37℃ for 30 minutes, before being analysed via flow cytometry.

**SA-β-gal Staining**

SA-β-Gal activity was observed in various samples using the SA-β-gal staining kit. Briefly, after cultivation for a certain time, MSCs were fixed with 4% paraformaldehyde for 15 minutes at 25℃. The cells were washed thrice with PBS before the addition of SA-β-gal staining solution, and incubated overnight at 37°C without a CO_2_ atmosphere. The treated samples were photographed using a light microscope (MVX10, Olympus, Japan), and the percentage of SA-β-gal staining was determined using ImageJ software.

**Cellular Immunofluorescence Staining**

The p21 protein, a marker of senescence, was detected by immunofluorescent staining. The cells were fixed with 4% paraformaldehyde and treated with 0.1% Triton-100 for 10 minutes, followed by several washes with PBS. The samples were then soaked in 5% BSA for one hour, followed by the addition of a primary antibody and incubation at 4°C overnight. After the incubation, fluorescently labelled secondary antibodies were incubated for 1 h in the absence of light. Afterward, DAPI was added and left for 15 minutes. Observation of samples was by CLSM.

**Evaluation of the ability to differentiate *in vitro***

To study the regulatory effect of modified titanium on the differentiation ability of ageing MSCs, alkaline phosphatase (ALP) vitality, collagen secretion, extracellular matrix (ECM) mineralization estimation, oil red O level, and expression of genes and proteins related to osteogenic differentiation and adipogenic differentiation were detected.

ALP vitality: MSCs were incubated with modified titanium in 24-well plates for 4 and 7 days. MSCs were fixed with 4% paraformaldehyde for 15 minutes, washed three times with PBS, and analysed qualitatively using the BCIP/NBT alkaline phosphatase detection kit and quantitatively using the p-nitrobenzene phosphatase detection kit.

Collagen secretion: MSCs in 24-well plates were incubated with modified titanium for 14 days and fixed with 4% paraformaldehyde. Images were then captured under a stereomicroscope using a Sirius Red staining solution. For quantitative analysis, the sample was immersed in the NaOH solution (1 mM) for 10 minutes at room temperature. Finally, the OD540nm was measured with the supernatant.

ECM mineralization estimation: MSCs were stained with alizarin red sodium solution (ARS) after 14 and 21 days of culture with different samples. Quantitative analysis of ARS was then performed. First, the sample was incubated in a 10% v/v acetic acid solution for 30 minutes. The extracted cell suspension was then treated in a water bath at 85°C for 10 minutes and the supernatant was obtained after centrifugation. Finally, the absorbance was measured at OD405nm after the addition of 10% ammonium hydroxide solution in the same volume as the supernatant.

Oil Red O level detection: After 14 and 21 days of induction, Oil Red O staining was performed using a kit to identify lipid droplets. The proportion of lipid droplets was calculated using Image J software.

Real-Time PCR：Total cellular RNAs were isolated from MSCs cultured for a specified time using a total RNA extraction kit (Tiangen, China) and then reverse transcribed into cDNA using a PrimeScript RT kit (Takara, Japan). All primer sequences used were shown in Supplementary Table 2 and Supplementary Table 3 (Support Information). Real-time PCR (qPCR) was used to quantify the expression of osteogenic-related genes (OCN, Runx2, ALP, OPN, COLI) and adipogenic-related genes (PPARγ, Cebpa, Lpl, Fabp4, Fasn). Detection was performed using the CFX96 Real Time System (Bio-Rad, USA).

ELSA assay: After a set period of co-culture, the supernatant from each selected sample was collected for ELISA detection to measure the secretion of proteins associated with the experiment.

**Osteoclast Differentiation.**

The RAW264.7 cells were inoculated with different samples in 24-well plates and incubated with medium containing RANKL (50 ng/mL) and M-CSF (20 ng/mL) for induction of osteoclast differentiation. TRAP assay kits were used to detect TRAP activity in different samples after 3 and 7 days of culture. The expression of osteoclast-related genes was quantified by qPCR after total RNA extraction. All primer sequences used were shown in Supplementary Table 4 (Support Information). Finally, cell morphology was observed by CLSM using rhodamine-phalloidin and DAPI staining after a period of culture.

**Experiment *in vivo***

***In vivo* ROS detection**

The levels of ROS in the vicinity of the modified titanium were observed using dihydroethidine (DHE). Briefly, First, DHE is dissolved in dimethyl sulfoxide and diluted with 5 % Tween 80, 5 % polyethylene glycol 400 and 0.9 % sodium chloride prior to intravenous injection. After 7 days of feeding, rats were euthanised, femurs removed and fixed by rapid decalcification. After being embedded in paraffin, the sections were processed and recorded under a fluorescence microscope.

**Establishment of the model OVX and implantation surgeries.**

All in vivo animal procedures were approved by the animal welfare and ethical Committee of Chongqing University (CQU-IACUC-RE-202205-001). Sprague Dawley rats (SD rats,female, 13 months old) were acquired from Byrness Weil Biotech. Ltd. (Chongqing, China). Prior to implantation, 65 purchased female rats were randomized in two groups: ovariectomized on both sides (OVX, 55 rats) and ovariectomized altogether (sham, 10 rats). After 3 months of feeding under standard conditions, the osteoporosis model was successfully established using microcomputed tomography scanning (Micro-CT). All OVX SD rats were then randomly divided into 5 groups, with 6 rats in each group receiving a modified titanium implant. Briefly, OVX SD rats were anaesthetised by intraperitoneal injection of a moderate amount of 4% chloral hydrate. Then a bone defect model was created using medical instruments to implant the modified titanium implant. Finally, an appropriate amount of penicillin was injected into the muscle around the wound 1 day after surgery.

**Micro-CT assessment**

After implantation, OVX SD rats were fed for 2 months under standard experimental conditions, euthanised, femurs removed and fixed in 4% paraformaldehyde. New bone formation around modified titanium implants after surgical implantation was assessed by Micro-CT. After that, visualization software reconstructed the 3D model and measured bone volume percent (BV/TV), bone surface area/volume percent (BS/BV), trabecular number (Tb.N), trabecular thickness (Tb.Th) and trabecular spacing (Tb.Sp).

**Histological evaluation**

After the micro-CT, all the femur samples were collected and fixed in 4% paraformaldehyde and decalcified for 28 days. The samples were dehydrated, embedded and sectioned. H&E staining and Masson's trichrome staining were used to evaluate bone tissue regeneration. Simultaneously, immunohistochemical staining detected the expression of osteogenic, adipogenic, osteoclastic and senescence related proteins in bone tissue. Immunofluorescence staining was used to further investigate how implants regulated the expression of related proteins.

**RNA-seq**

The RNA-seq assay was used to investigate the effect of modified titanium on cell gene expression. Specifically speaking, After the material was incubated with the cells, the cells were collected and total RNA was extracted. The Illumina Novaseq 6000 sequencing platform of Magor Biomedical Technology Co. ltd (Shanghai, China) was used to obtain gene expression profiles. DEGseq2 software was used to analyse expression differences, and log2 fold change > |1| and padjust < 0.05 were considered as differentially expressed genes (DEGs). To further elucidate the biological significance of the differential genes, GO, KEGG and protein interaction network analyses were performed using Cytoscape software and the String database.

**Statistical analysis**

The experimental data were presented as the mean ± standard deviation (SD). ANOVA and Student's t-test were performed for statistical significance of the data using GraphPad Prism (version 10) software. P values less than 0.05 were regarded as statistically significant. The * and ** represent P<0.05 and P<0.01, respectively.

Figure S1.


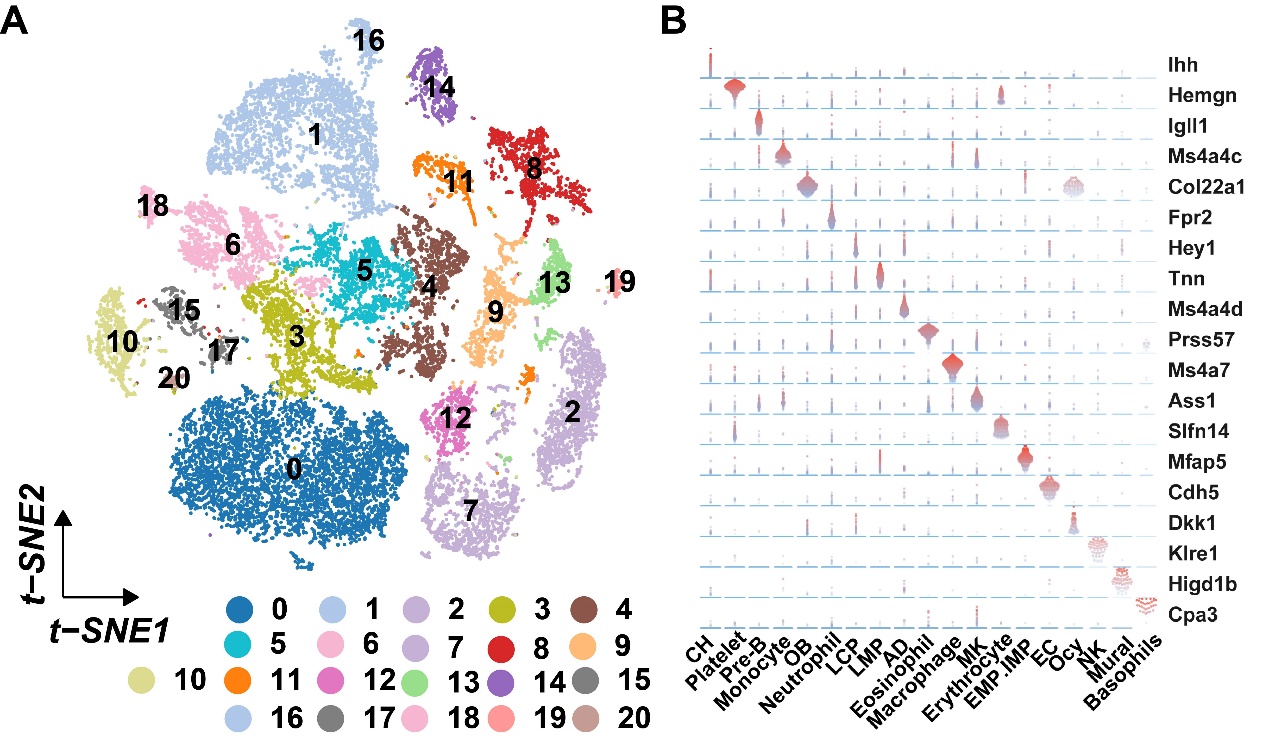


**Figure S1.** (A) The tSNE plot of 24049 bone marrow cells isolated from bone marrow at 1, 3 and 16 months. (B) Violin plots of cluster-specific makers.

Figure S2.


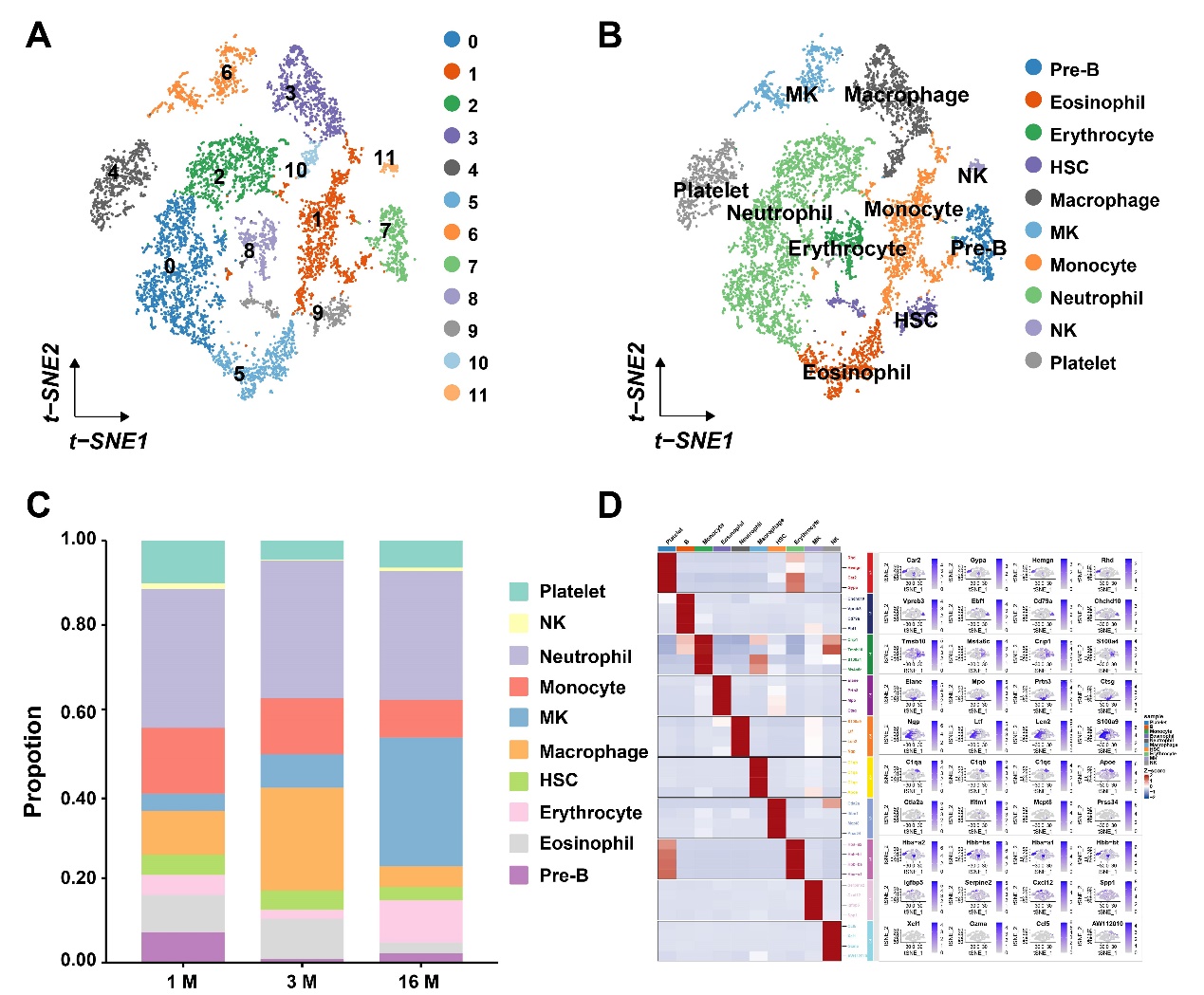


**Figure S2.** (A) The tSNE plot of 8265 hematopoietic lineage cells isolated from bone marrow at 1, 3 and 16 months. (B) Annotation of cell subpopulations in hematopoietic lineage cells at 1, 3, and 16 months of age. (C) Proportions of different cell subsets in hematopoietic lineage cells derived from 1, 3 and 16 months of age. (D) The marker genes and their average expression levels in hematopoietic lineage cell subsets.

Figure S3.


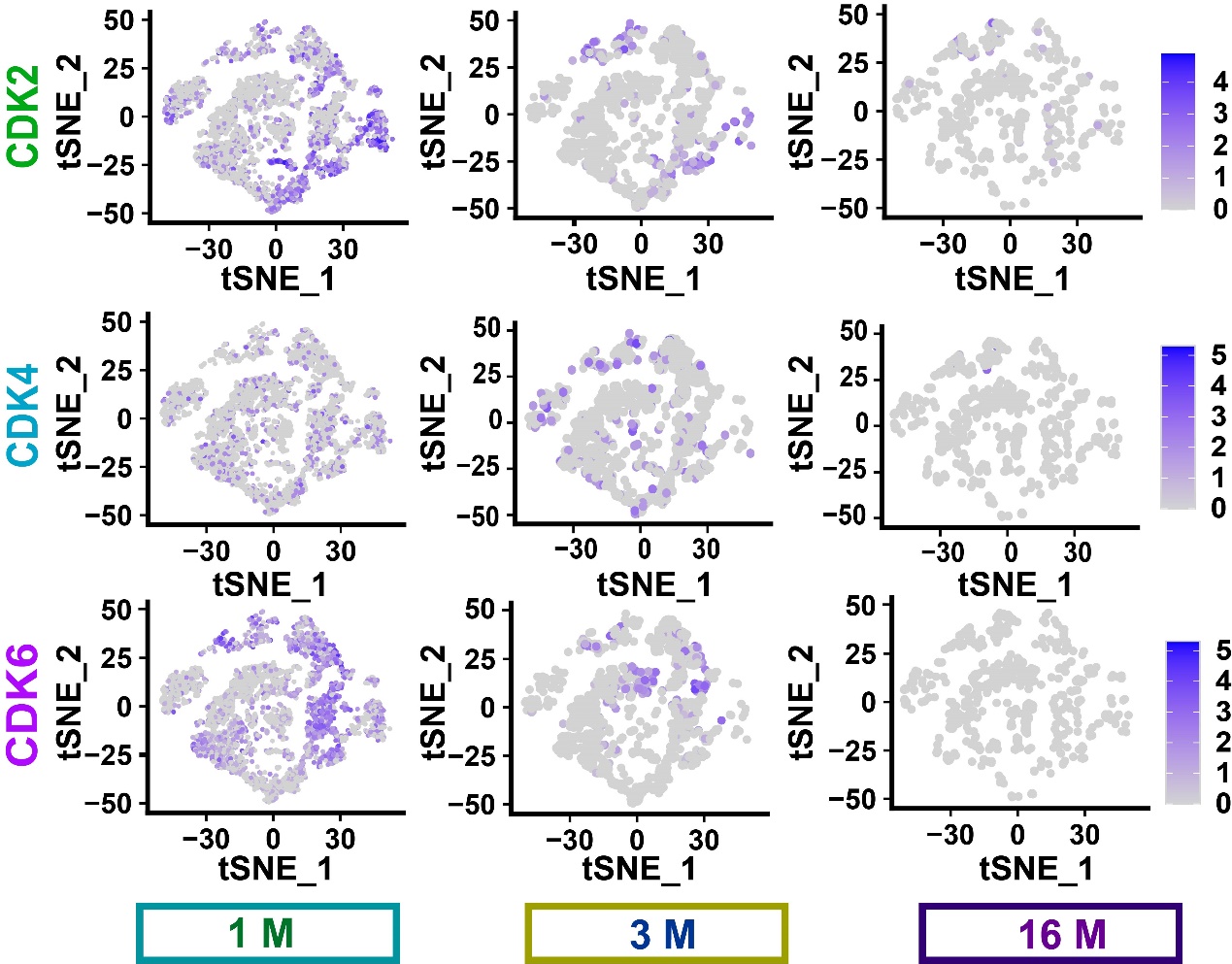


**Figure S3.** (A) The expression levels of CDK2, CDK4 and CDK6 in bone marrow hematopoietic lineage cells were measured at 1, 3 and 16 months.

Figure S4.


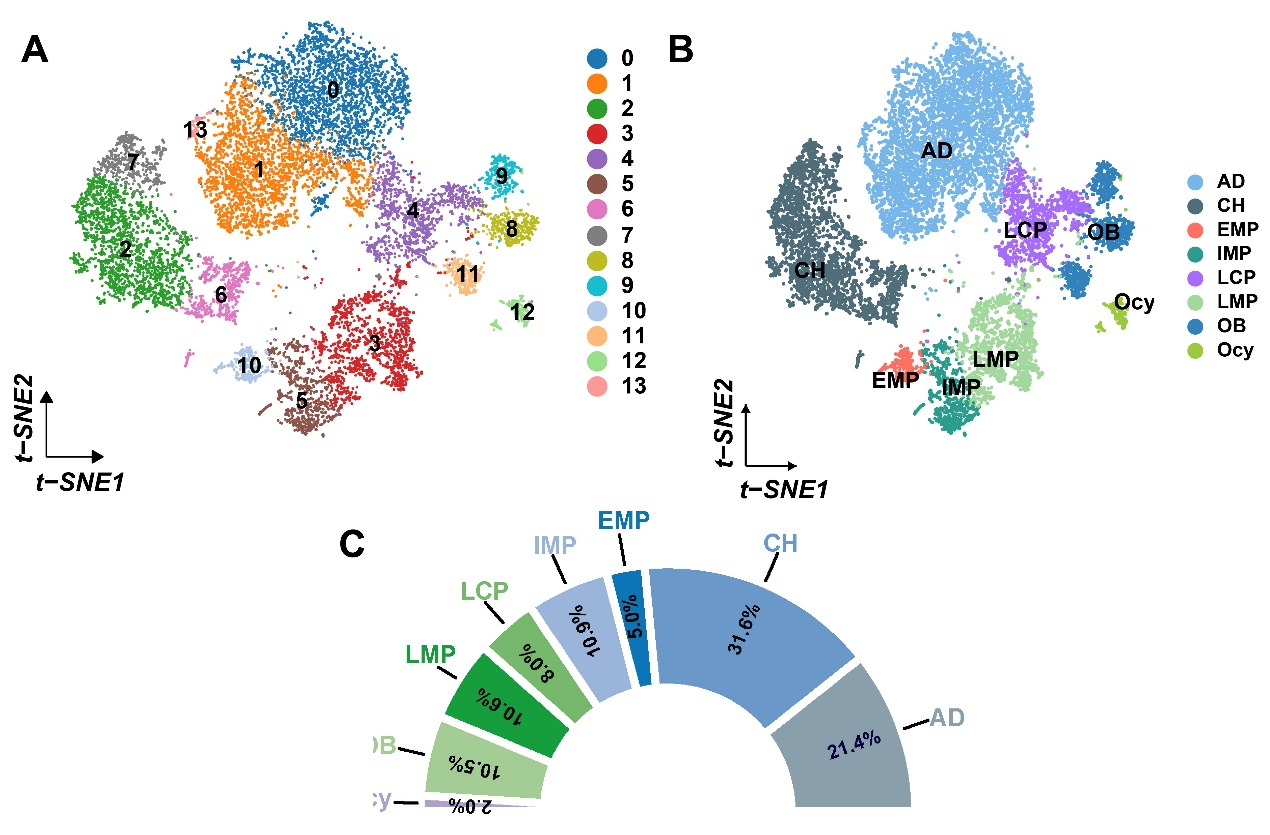


**Figure S4.** (A) The tSNE plot of 13938 bone marrow mesenchymal lineage cells isolated from bone marrow at 1, 3 and 16 months. (B) Annotation of cell subpopulations in bone marrow mesenchymal lineage cells at 1, 3, and 16 months of age. (C) The proportion of cell subsets in bone marrow mesenchymal lineage cells.

Figure S5.


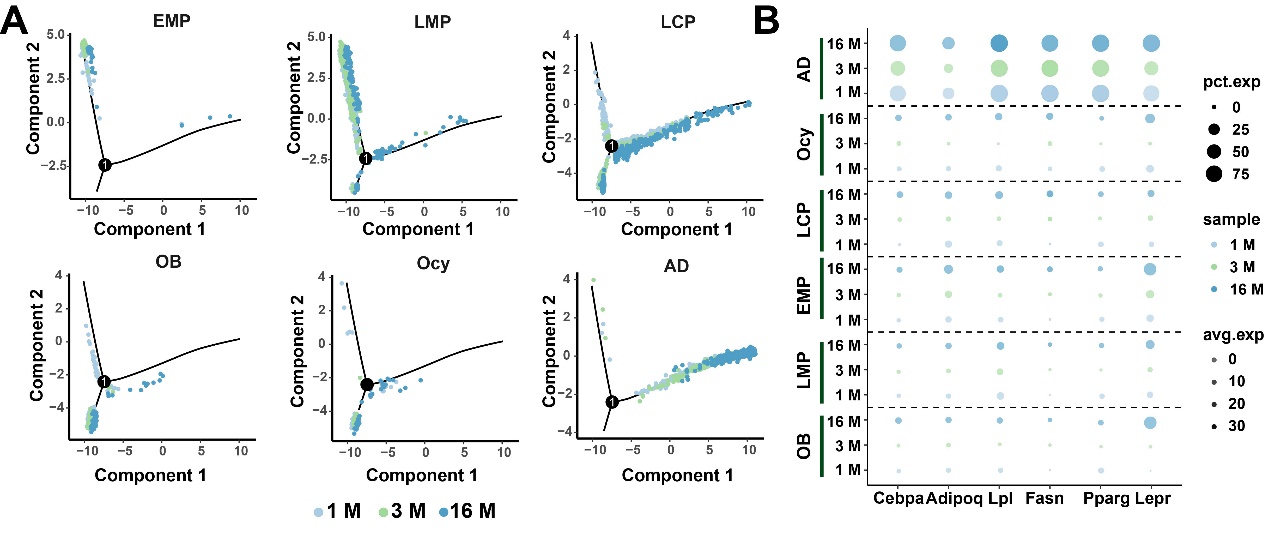


**Figure S5.** (A) Monocle trajectory plots are separated based on age groups and Seurat clusters; (B) Dotplot of genes associated with adipogenic differentiation in Seurat clusters across different age groups.

Figure S6.


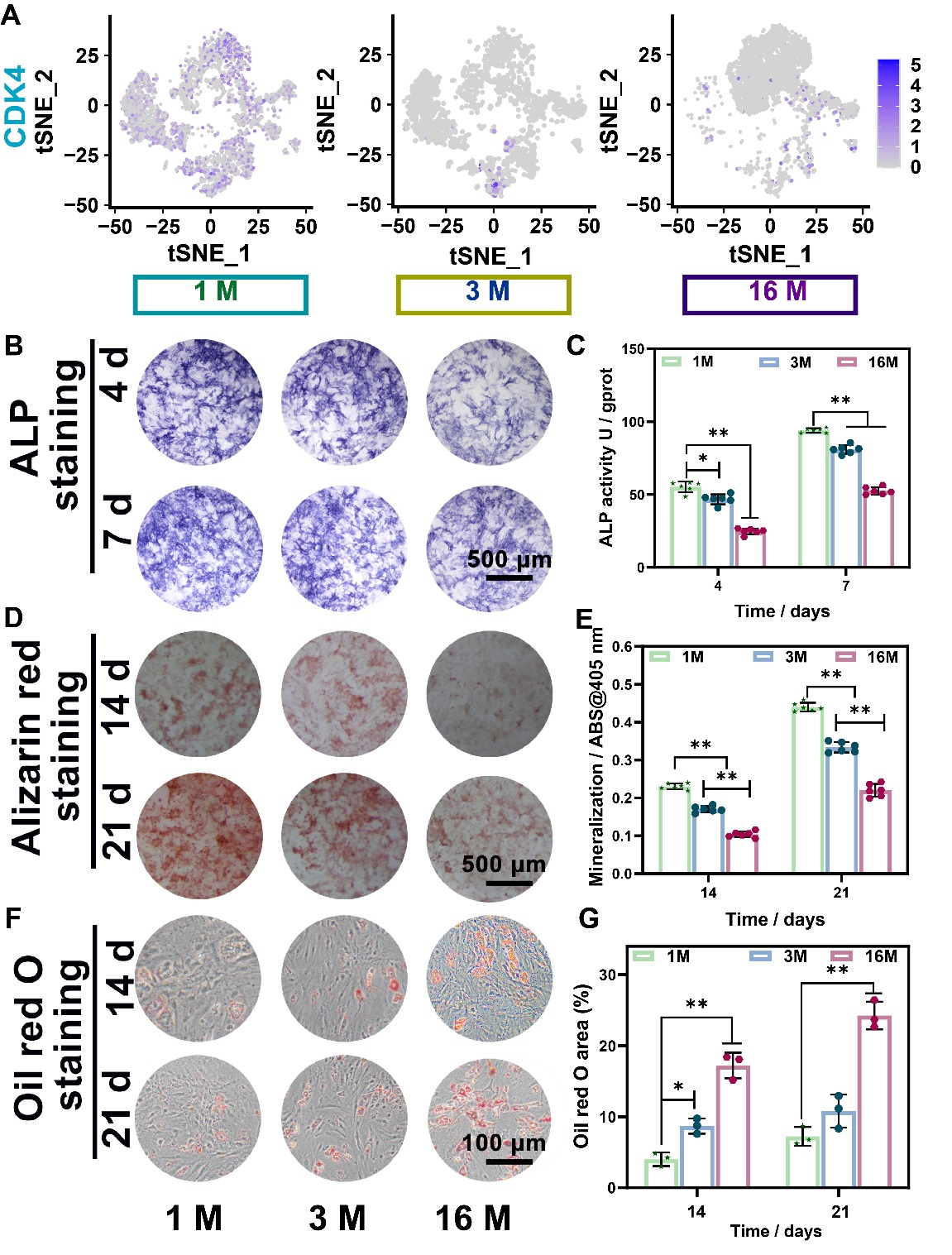


**Figure S6.** (A) The expression level of CDK4 in bone marrow mesenchymal cells was detected at 1, 3 and 16 months. Qualitative (B) and quantitative (C) analysis of ALP (n=6). Qualitative (D) and quantitative (E) analysis of mineralisation capacity (n=6). Qualitative (F) and quantitative (G) analysis of lipid formation levels (n=3). Data were presented as mean values ± standard deviations (SD); error bars = SD. **p* <0.05, ***p* <0.01.

Figure S7.


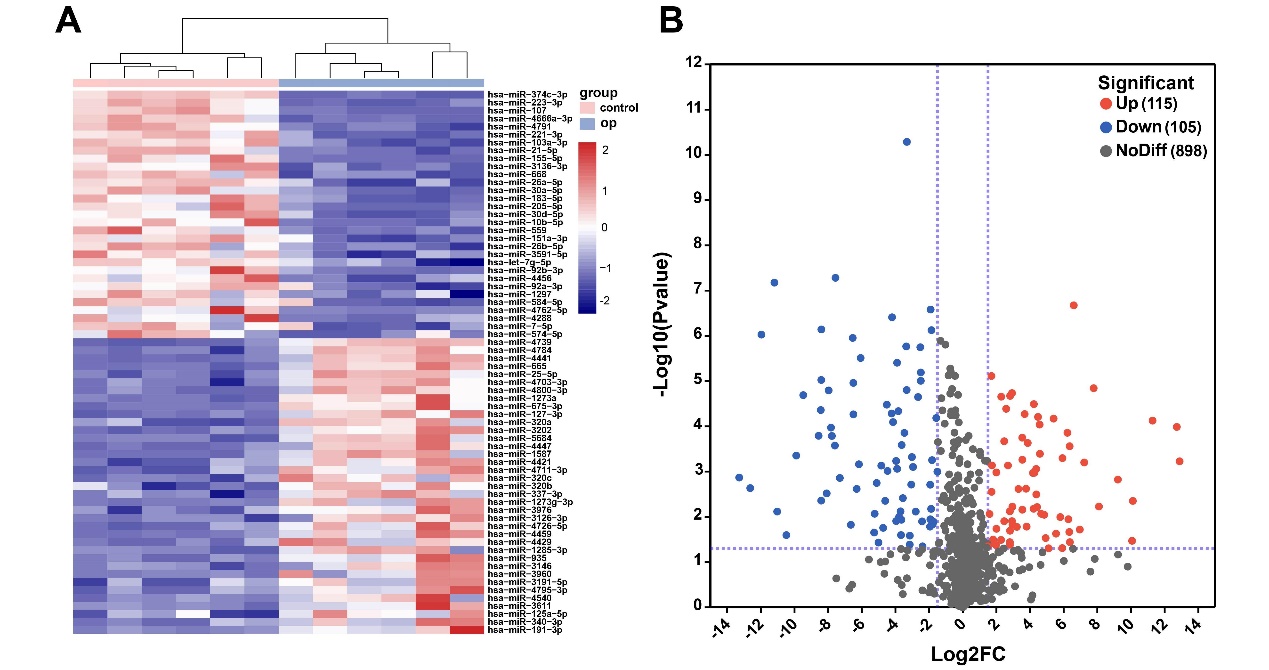


**Figure S7.** (A) Differential miRNA heatmaps. (B) Differential miRNA volcano map.

Figure S8.


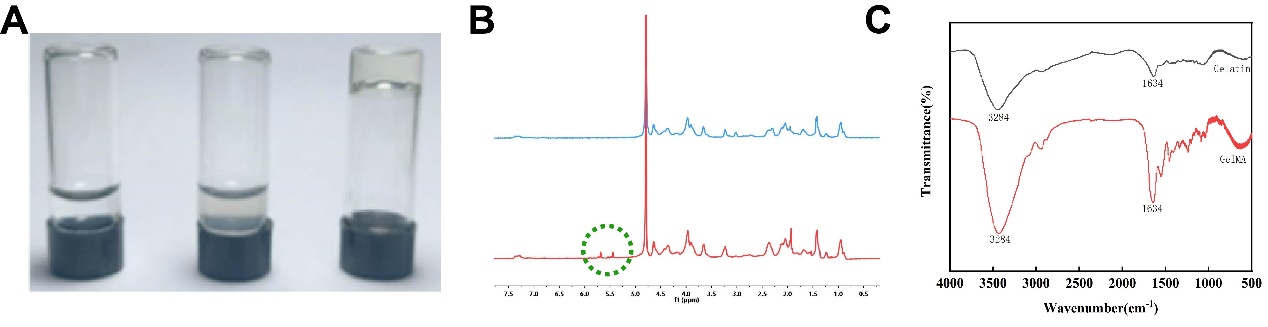


**Figure S8.** (A) GelMA, carboxylated chitosan and a hydrogel photograph of the mixture of the two. (B) ^1^H NMR of Gelatin and GelMA. (C) FTIR spectra of Gelatin and GelMA.

Figure S9.

­­
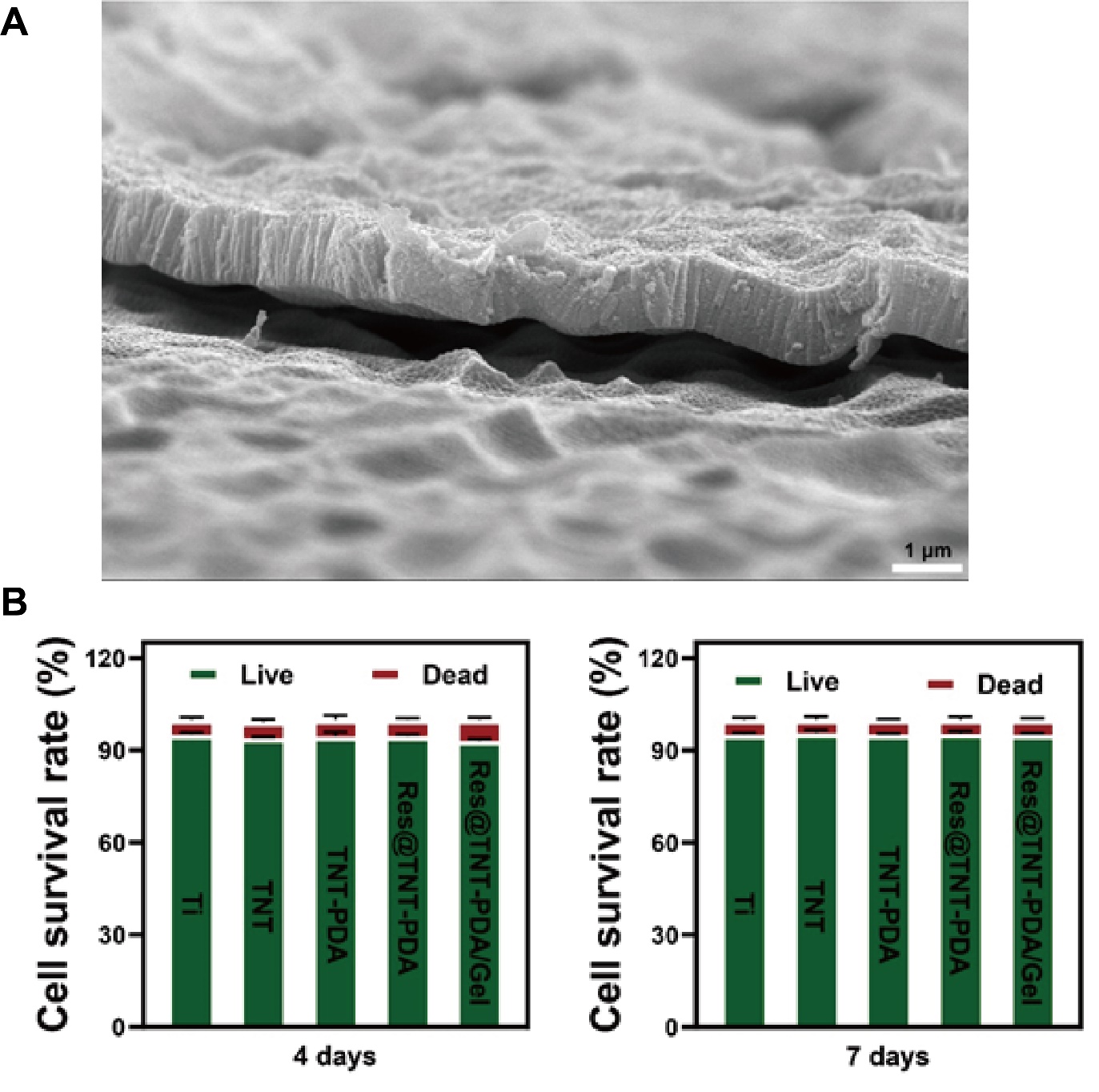


**Figure S9.** (A) The thickness image of TNT. (B) Quantitative analysis of live/dead staining of senescent bone marrow mesenchymal stem cells on Ti, TNT, TNT- pda, Res@TNT-PDA and Res@TNT-PDA/Gel was observed after 4 days and 7 days.

Figure S10.


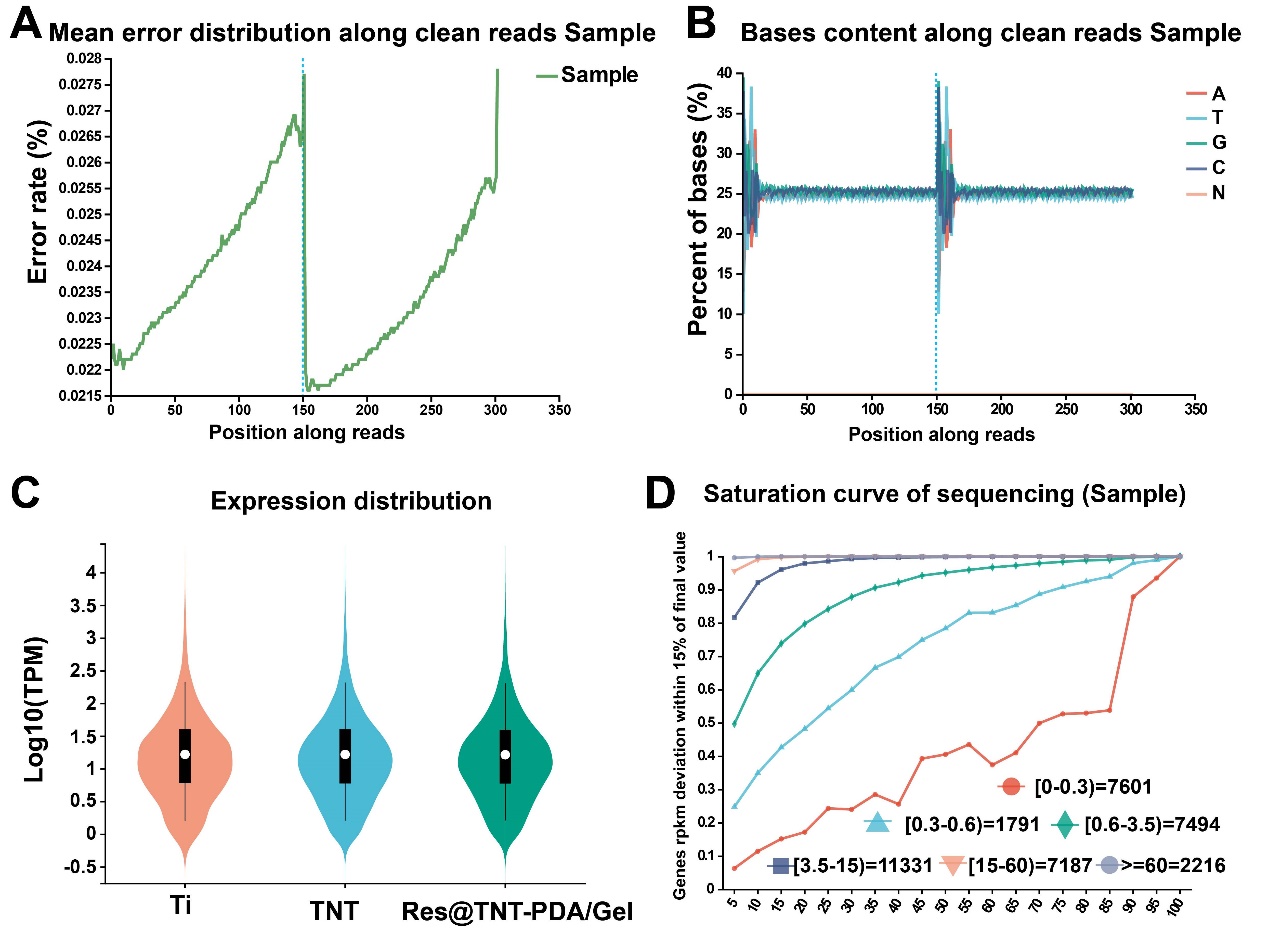


Figure S10. Transcriptomic analysis of differential gene expression in different groups of samples. Mean error distribution (A), bases content (B), gene expression distribution (C), and saturation curve (D) of the Transcriptomic analysis

Figure S11.


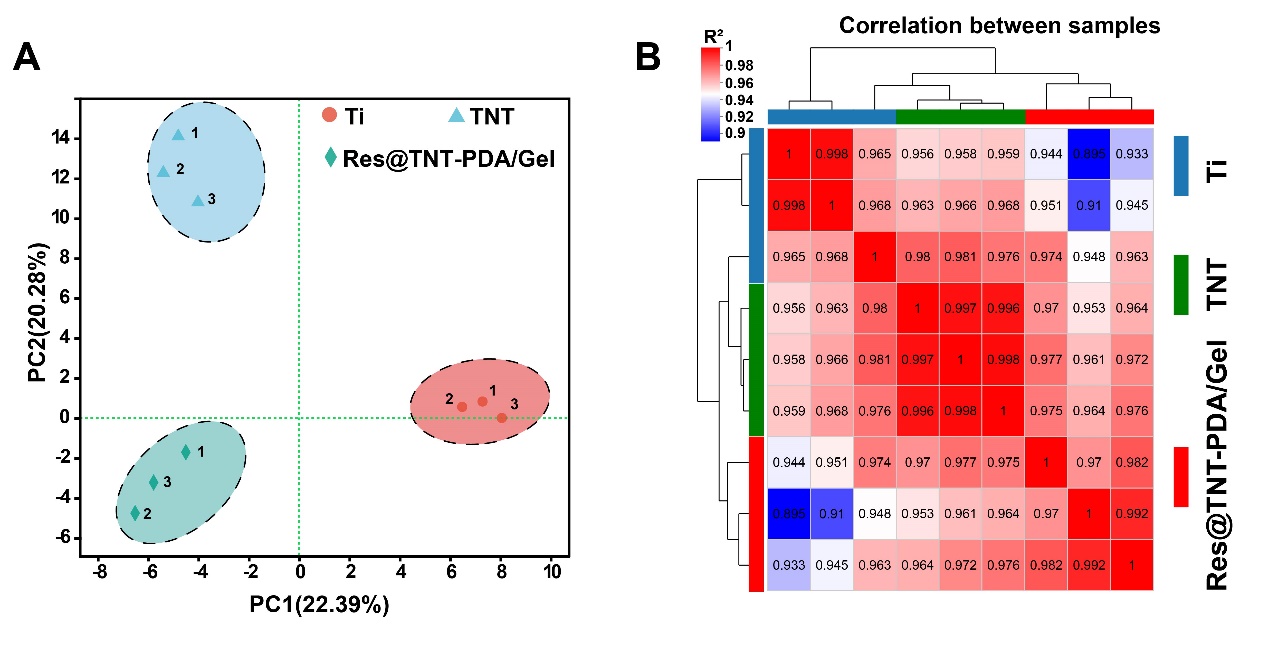


**Figure S11.** (A) Principal component analysis (PCA) among all experimental samples. (B) The correlation analysis heatmap between all samples.

Figure S12.


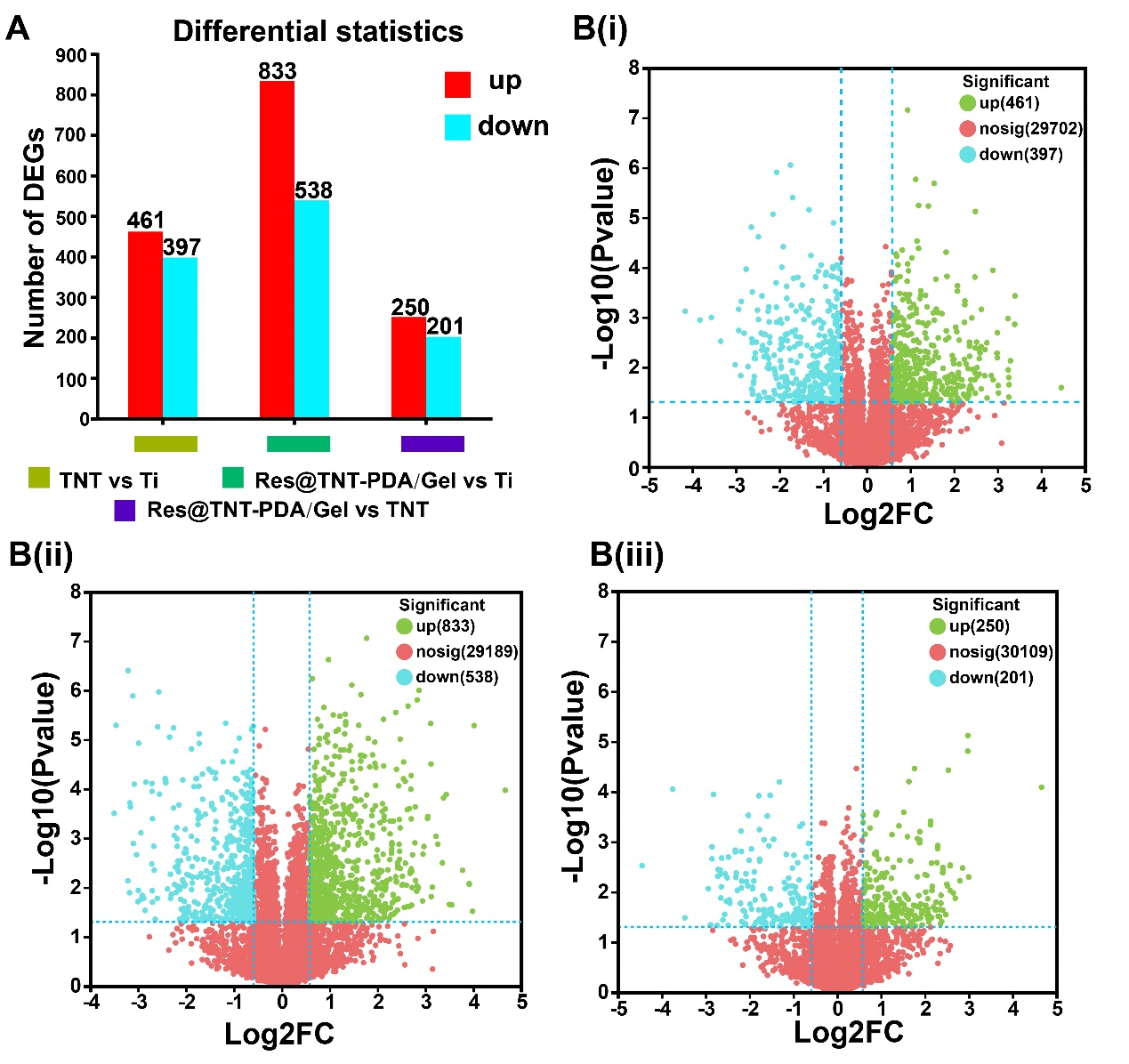


Figure S12. (A) Quantitative analysis of differentially expressed genes between samples. (B) Volcanic map of differential genes. Green dots represent up-regulated genes, blue dots represent down-regulated genes and red dots represent genes with no significant difference in measurement.

Figure S13.


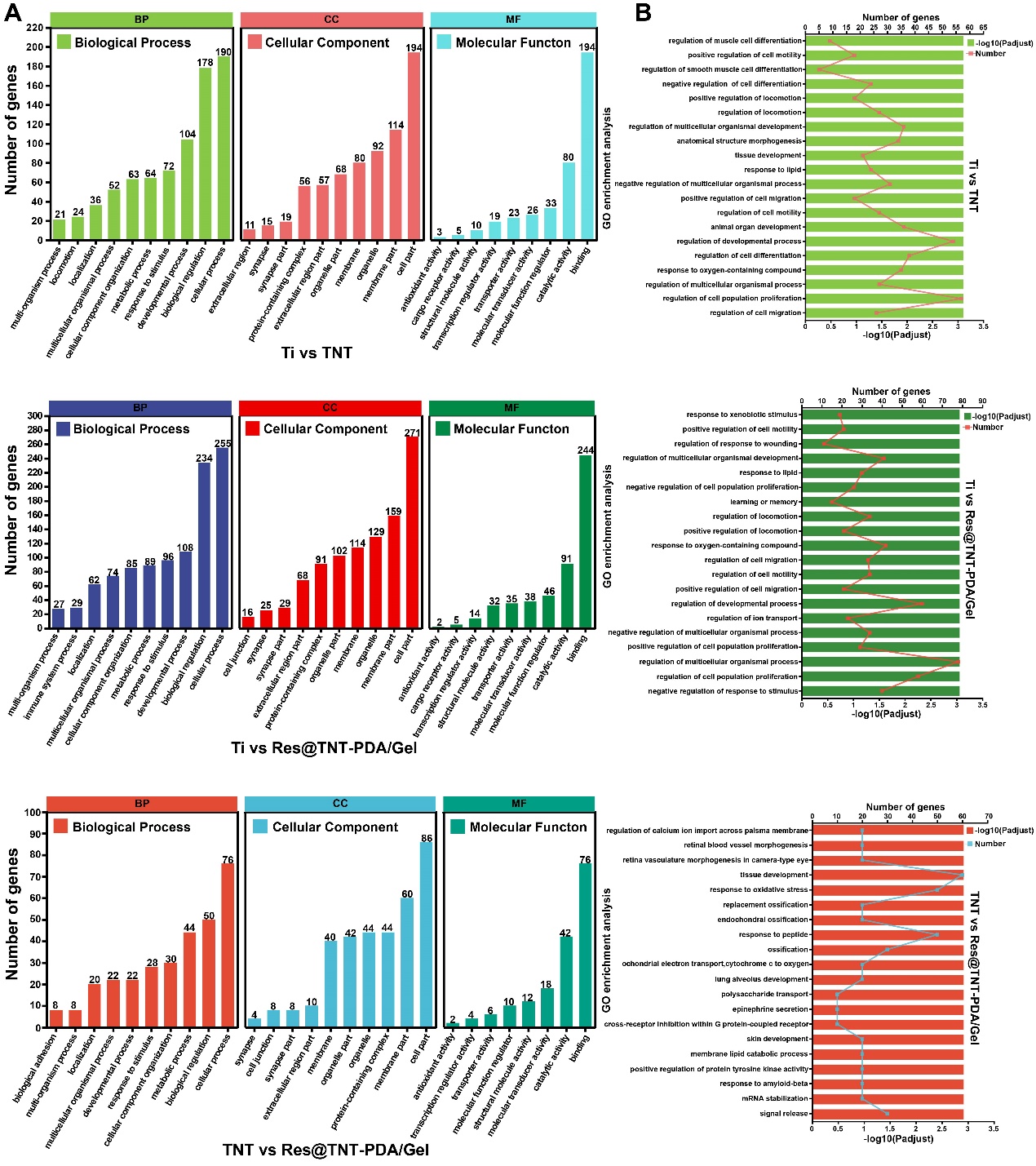


**Figure S13.** GO enrichment analysis between pairwise groups. (A) Annotations analysis of GO item between pairwise group, Ti vs TNT, Ti vs Res@TNT-PDA/Gel, TNT vs Res@TNT-PDA/Gel. (B) GO enrichment analysis of different pairwise group, Ti vs TNT, Ti vs Res@TNT-PDA/Gel, TNT vs Res@TNT-PDA/Gel.

Figure S14.


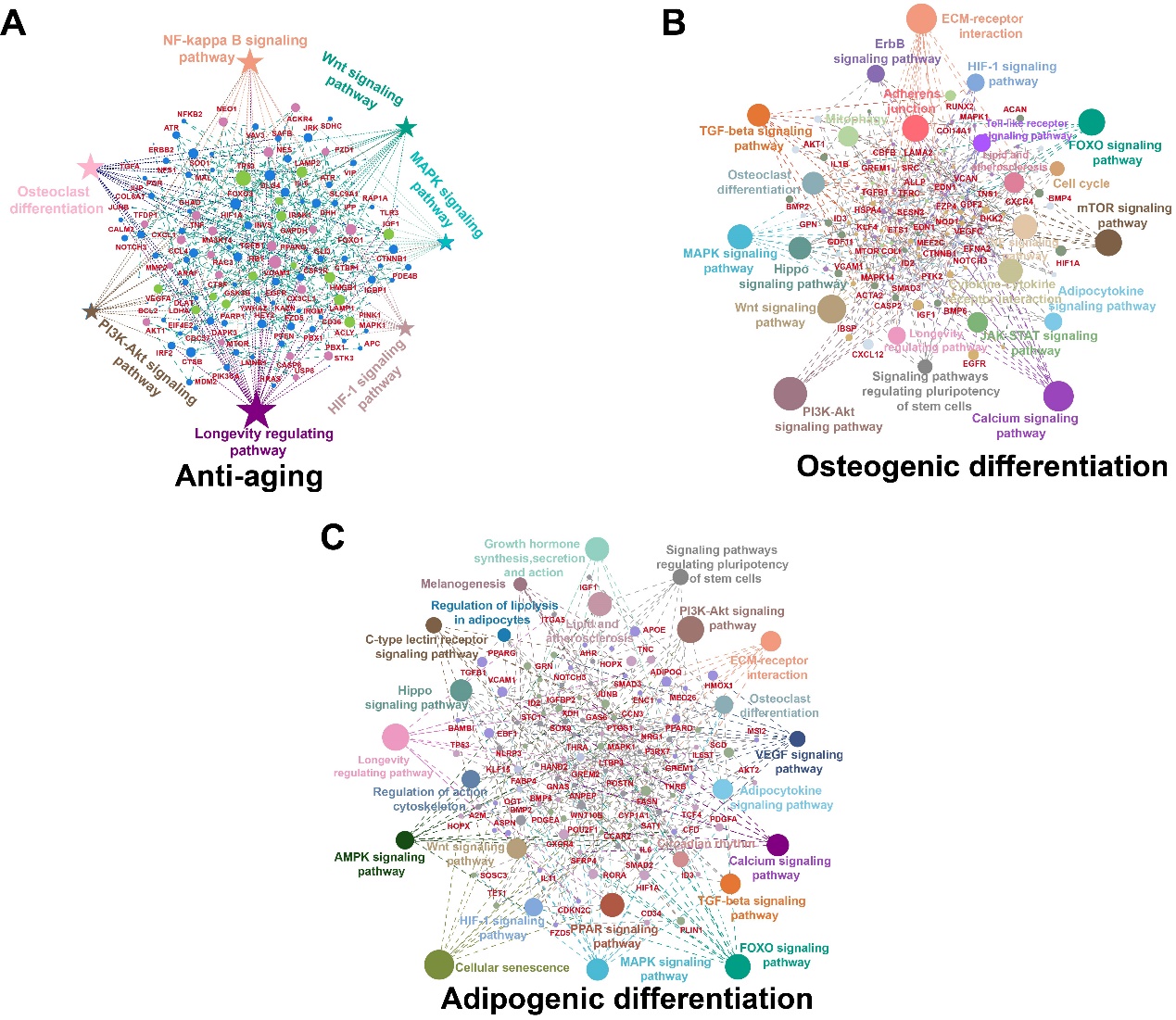


**Figure S14.** KEGG enrichment analysis and protein interaction network analysis. (A) KEGG enrichment analysis and protein interaction network analysis of the differential genes associated with anti-aging. (B) KEGG enrichment analysis and protein interaction network analysis of the differential genes associated with osteogenic differentiation. (C) KEGG enrichment analysis and protein interaction network analysis of the differential genes associated with adipogenic differentiation.

Figure S15.


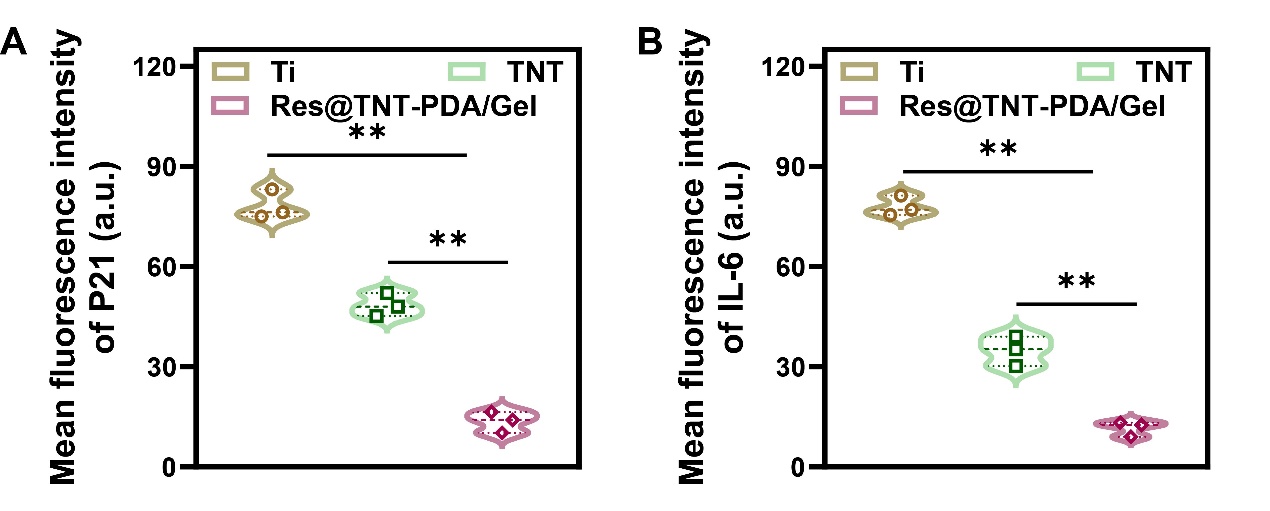


**Figure S15.** Quantification of relative fluorescence intensity of P21. Quantification of relative fluorescence intensity of IL-6.

Table S1.

**Supplementary Table 1.** Full cell name and abbreviation.

|  | The full name of the cell | Cell name abbreviation |
| --- | --- | --- |
| 1 | Early mesenchymal progenitors | EMP |
| 2 | Intermediate mesenchymal progenitors | IMP |
| 3 | Late mesenchymal progenitors | LMP |
| 4 | Lineage committed progenitors | LCP |
| 5 | Osteoblast | OB |
| 6 | Osteocyte | Ocy |
| 7 | Adipocyte | AD |
| 8 | Chondrocyte | CH |
| 9 | Hematopoietic stem cells | HSC |
| 10 | Pre-B cells | Pre-B |
| 11 | Megakaryocyte | MK |
| 12 | Macrophages | Macrophage |
| 13 | Neutrophil cells | Neutrophil |
| 14 | Natural killer cells | NK |
| 15 | Eosinophil cells | Eosinophil |
| 16 | Erythrocyte | Erythrocyte |
| 17 | Platelet | Platelet |
| 18 | Endothelial cells | EC |
| 19 | Mural cells | Mural |

Table S2.

**Supplementary Table 2.** Primers for qPCR analysis (species, SD Rat).

| **Genes** | **Primers sequences** |
| --- | --- |
| GAPDH | 5’- GGTGCTGAGTATGTCGTGGAGTC -3’ |
|  | 5’- CATTGCTGACAATCTTGAGGGAG -3’ |
| ALP | 5’- CACTATGTCTGGAACCGCACTG -3’ |
|  | 5’- AAGCCTTTGGGATTCTTTGTCA -3’ |
| OCN | 5’- AGATTGTTGGGGCACAAGGT -3’ |
|  | 5’- CCTTCAGCAGGGAAACCGAT -3’ |
| OPN | 5’- CCTTCAGCAGGGAAACCGAT -3’ |
|  | 5’- CAGGCTGGCTTTGGAACT -3’ |
| RUNX2 | 5’- GCCGTAGAGAGCAGGGAAGAC -3’ |
|  | 5’- CTGGCTTGGATTAGGGAGTCAC -3’ |
| COLI | 5’- CCTGAGCCAGCAGATTGA -3’ |
|  | 5’- TCCGCTCTTCCAGTCAG -3’ |

Table S3.

**Supplementary Table 3.** Primers for qPCR analysis (species, SD Rat).

| **Genes** | **Primers sequences** |
| --- | --- |
| GAPDH | 5’- AGGTCGGTGTGTGAACGGATTTG -3’ |
|  | 5’- TGTAGACCATGTAGTTGAGGTCA -3’ |
| LPL | 5’- CACAGTGGCTGAAAGTGAGAACA -3’ |
|  | 5’- CCAGCGGAAGTAGGAGTCGTT -3’ |
| CEBPA | 5’- CAAGGGCTTGGCTGGTCC -3’ |
|  | 5’- GTTGCGTTCCCGCCGCCGTAC -3’ |
| PPARγ | 5’- CTGCGTCCCCGCCTTATTA -3’ |
|  | 5’- CCCACAGACTCGGCACTCG -3’ |
| FABP4 | 5’- CGTAGAAGGGGACTTGGTCGT -3’ |
|  | 5’- TTCCTGTCATCTGGGGTGATTT -3’ |
| FASN | 5’- CTGTTATCACCCGACTTCCTCTG -3’ |
|  | 5’- TGCTGAATACGACCACGCACTA -3’ |

Table S4.

**Supplementary Table 4.** Primers for qPCR analysis (species, mouse).

| **Genes** | **Primers sequences** |
| --- | --- |
| β-Actin | 5’- ACAGCAGTTGGTTGGAGCAA -3’ |
|  | 5’- ACGCGACCATCCTCCTCTTA -3’ |
| CTSK | 5’- GAAGAAGACTCACCAGAAGCAG -3’ |
|  | 5’- TCCAGGTTATGGGCAGAGATT -3’ |
| OSM | 5’- CCCGGCACAATATCCTCGG -3’ |
|  | 5’- TCTGGTGTTGTAGTGGACCGT -3’ |
| NFATC1 | 5’- GGAGAGTCCGAGAATCGAGAT -3’ |
|  | 5’- TTGCAGCTAGGAAGTACGTCT -3’ |
| RNAK | 5’- CCAGGAGAGGCATTATGAGCA -3’ |
|  | 5’- ACTGTCGGAGGTAGGAGTGC -3’ |
| ACP5 | 5’- CCATTGTTAGCCACATACGG -3’ |
|  | 5’- CACTCAGCACATAGCCCACA -3’ |
